# Porous barriers? Assessment of gene flow within and among sympatric long‐eared bat species

**DOI:** 10.1002/ece3.4714

**Published:** 2018-12-07

**Authors:** Tommy Andriollo, Sohrab Ashrafi, Raphaël Arlettaz, Manuel Ruedi

**Affiliations:** ^1^ Department of Mammalogy and Ornithology Natural History Museum of Geneva Geneva Switzerland; ^2^ Section of Biology, Faculty of Sciences University of Geneva Geneva Switzerland; ^3^ Department of Environmental Sciences, Faculty of Natural Resources University of Tehran Karaj Iran; ^4^ Division of Conservation Biology, Institute of Ecology and Evolution University of Bern Bern Switzerland

**Keywords:** Chiroptera, cryptic species, gene flow, hybridization, population genetics, reproductive barriers

## Abstract

Species are the basic units for measuring biodiversity and for comprehending biological interactions. Yet, their delineation is often contentious, especially in groups that are both diverse and phenotypically conservative. Three cryptic species of long‐eared bats, *Plecotus auritus*, *P. austriacus,* and *P. macrobullaris*, co‐occur over extensive areas of Western Europe. The latter is a fairly recent discovery, questioning the overall diversity of the entire *Plecotus* complex. Yet, high morphological and acoustic similarities compromise the reliable identification of long‐eared bats in the field. We postulate that such extensive phenotypic overlap, along with the recurrent observation of morphologically intermediate individuals, may hide rampant interspecific hybridization. Based on a geographic sampling centered on areas of sympatry in the Alps and Corsica, we assessed the level of reproductive isolation of these three *Plecotus* species with mitochondrial and nuclear markers, looking at both inter‐ and intraspecific genetic population structuring. No sign of hybridization was detected between these three species that appear well separated biologically. Genetic structuring of populations, however, reflected different species‐specific responses to environmental connectivity, that is, to the presence of orographic or sea barriers. While the Alpine range and the Ligurian Sea coincided with sharp genetic discontinuities in *P. macrobullaris* and *P. austriacus*, the more ubiquitous *P. auritus* showed no significant population structuration. There were clear phylogeographic discrepancies between microsatellite and mitochondrial markers at the intraspecific level, however, which challenges the reliance on simple barcoding approaches for the delineation of sound conservation units.

## 1 introduction

### 1.1 Barriers to gene flow

Among the tens of species concepts proposed in the literature, the most widely used is the biological species concept, coined by Mayr (1963) who defined species as “groups of actually or potentially interbreeding natural populations, which are reproductively isolated from other such groups.” However, it is now accepted that many well‐recognized species can interbreed to some extent without implying necessarily the lumping of the involved taxa (Harrison & Larson, 2014; Kane et al., 2009). Striking examples of cross‐species gene flow occur between deeply divergent lineages such as across distinct genera of birds (Grant & Grant, 1992), fishes (Amini, Zamini, & Ahmadi, 2007; Bartley, Rana, & Immink, 2000) or flowering plants (Knobloch, 1972). Conversely, a growing number of examples show that porous reproductive barriers are not preventing speciation from taking place, implying that speciation with gene flow is a more common phenomenon than previously thought (Nosil, 2008; Petit & Excoffier, 2009).

Porous interspecific barriers are reported in mammals and birds, with an estimated 10% of species showing hybridization and interspecific introgression (Mallet, 2005). Yet, in the speciose order of Chiroptera, accounting for 20% of all mammal species (Burgin, Colella, Kahn, & Upham, 2018), cases of hybridization appear to be rare. For instance, the family Vespertilionidae contains about 500 species but only includes very few well‐documented cases of interspecific hybrids (Afonso, Goydadin, Giraudoux, & Farny, 2017; Berthier, Excoffier, & Ruedi, 2006; Centeno‐Cuadros et al., 2017), whereas cases of historical events of gene introgression exemplified by cytonuclear discrepancies are more common (Artyushin, Bannikova, Lebedev, & Kruskop, 2009; Baird, Hillis, Patton, & Bickham, 2008; Kuo et al., 2015; Morales & Carstens, 2018; Morales, Jackson, Dewey, O'meara, & Carstens, 2017; Platt et al., 2017; Trujillo, Patton, Schlitter, & Bickham, 2009; Vallo, Benda, Červený, & Koubek, 2013). However, it is possible that contemporary interspecific hybridization is underestimated, since most of the taxonomic studies conducted on bats usually rely only on mitochondrial genes for species‐level recognition (Baker & Bradley, 2006; Clare, Lim, Engstrom, Eger, & Hebert, 2007; Francis et al., 2010). Indeed, due to its uniparental and clonal mode of inheritance, this type of genetic marker is not well‐suited to identify individuals of hybrid origin (Berthier et al., 2006) and the use of nuclear, biparentally inherited markers is often necessary to assess the porosity of interspecific barriers in more detail (Ballard & Whitlock, 2004).

Beyond reproductive barriers, species are also confronted to topographical barriers that can influence the genetic architecture of populations. In particular, major landscape features such as mountain ranges or large expanses of open water may have served as important barriers to dispersal in bats (Castella, Ruedi, & Excoffier, 2001; Dàvalos, 2005; Juste et al., 2004). This is particularly true in European biotas which experienced multiple events of range contraction and expansion caused by glacial cycles (Hewitt, 1999). Southern glacial refugia, such as the Iberian, Apennine, and Balkan peninsulas, retained genetic diversity and allowed population differentiation during phases of allopatry, whereas topographical barriers such as the Alps or the Pyrenees constrained recolonization routes from those refugia (Çoraman, Furman, Karataş, & Bilgin, 2013; Hewitt, 2000). Predicting the effect of these barriers on gene flow can be tricky, since co‐distributed species with distinct ecological needs may show different responses to the same landscape elements, depending on their ability to cross those (Engler, Balkenhol, Filz, Habel, & Rödder, 2014; Zancolli, Rödel, Steffan‐Dewenter, & Storfer, 2014). For instance, large expanses of water can limit drastically the dispersal of some bat species, while they have minor effects on others (García‐Mudarra, Ibañez, & Juste, 2009). Likewise, the Alps have been shown to delimit major genetic pools in certain bat species (Ruedi et al., 2008; Wright et al., 2018) when others disperse extensively through this range (Rebelo et al., 2012).

### 1.2 Model species

Delimitation of species boundaries in long‐eared bats of the genus *Plecotus* has been challenging for decades. Because of their very similar external morphology, a single species was considered to occur in Europe until a second one was raised to species level by Bauer (1960) and Lanza (1960). More recently, with the use of genetics, no less than three species were further added to the European fauna (Kiefer & Veith, 2002; Mucedda, Kiefer, Pidinchedda, & Veith, 2002; Spitzenberger, Pialek, & Haring, 2001). In the Alps, where the three species *P. auritus*, *P. austriacus* and *P. macrobullaris* coexist, their discrimination in the field on the basis of external morphology remains difficult (Ashrafi, Bontadina, Kiefer, Pavlinić, & Arlettaz, 2010). Even the examination of cranial characters showed the existence of intermediate specimens with conflicting genetic and morphologic diagnoses (Andriollo & Ruedi, 2018). These discrepancies raise the question whether these three *Plecotus* species are reproductively isolated from each other or not. If interspecific barriers are porous, these inconsistencies could result from hybridization leading to individuals with atypical morphology or introgressed genes. Furthermore, since the degree of reproductive isolation is expected to increase with time since divergence (Edmands, 2002), we hypothesized that hybridization should be more likely between the two more closely related species (*P. auritus* and *P. macrobullaris*) than with *P. austriacus*, which originated from a much older divergence event (Spitzenberger, Strelkov, Winkler, & Haring, 2006).

Since two or more of these morphologically similar species of long‐eared bats occur in sympatry over extensive areas in continental Europe and Corsica (Andriollo & Ruedi, 2018; Courtois, Rist, & Beuneux, 2011; Gilliéron, Schönbächler, Rochet, & Ruedi, 2015; Rutishauser, Bontadina, Braunisch, Ashrafi, & Arlettaz, 2012; Tvrtković, Pavlinić, & Haring, 2005), they also offer the opportunity to compare the barrier effect of major landscape features on gene flow in each species in parallel. Indeed this geographical region includes two main potential topographic barriers to dispersal and gene flow, the Alps mountain range and the Ligurian Sea. Judging from their similar wing morphology, all three species of long‐eared bats are considered as poor long‐distance flyers (Entwistle, Racey, & Speakman, 1996). Ringing studies indeed show that they are highly sedentary species (Hutterer, Ivanova, Meyer‐Cords, & Rodrigues, 2005; Racey & Entwistle, 2003), although little is known about dispersal abilities in *P. macrobullaris* (Alberdi, Aihartza, et al., 2015a; Ashrafi et al., 2013). Accordingly, genetic structure of populations in these bats is generally pronounced and potentially highly affected by extrinsic factors such as geographic barriers to dispersal (Burland & Worthington‐Wilmer, 2001; Racey & Entwistle, 2003).

However, since the general ecology of these three species differs, for instance in terms of altitudinal preferences (Rutishauser et al., 2012), more specific predictions of the effect of topographic barriers can be formulated. *P. macrobullaris* is a typically alpine‐adapted species that is able to breed at altitudes of 2,000 m a.s.l. and higher (Alberdi, Garin, Aizpurua, & Aihartza, 2013; Anonymous, 2014b); the Alps should likely not be a major obstacle to dispersal, while the species might be more reluctant to cross intervening lowland areas. On the contrary, *P. austriacus* is a typical lowland species (Anonymous, 2014a; Arthur & Lemaire, 2015; Juste et al., 2008), and the reverse situation may likely prevail. Finally, *P. auritus* occurs across a wide altitudinal range in the Alps (Anonymous, 2014c; Hutson et al., 2008) and thus is expected to be somewhat intermediate regarding the effect of mountain barriers on gene flow. Predictions about the effect of sea channels on gene flow in these three species are more difficult to draw. On the one hand, *P. austriacus* seems little affected by sea barriers as it is found on most Mediterranean islands and as far as Madeira (Juste et al., 2004; Spitzenberger et al., 2006), whereas *P. auritus* or *P. macrobullaris* appear more reluctant to cross open water as they are found only on one or two of these islands (Hutson et al., 2008; Piraccini, 2016). We can thus predict that the Ligurian Sea should be a more effective barrier to gene flow for the latter two than for the former species.

We used a combination of nuclear and mtDNA markers on bats sampled in areas where those species co‐occur in strict sympatry to explore (a) their degree of reproductive isolation and (b) to which extent the Alpine range and Ligurian Sea constitute topographic barriers limiting gene flow among populations.

## 2 material and methods

### 2.1 Sampling design and assignation to major mtDNA lineages

To detect the presence of putative hybrids between the three *Plecotus* species, we sampled several areas where they have true chances to interbreed, that is, zones of strict sympatry. A total of 349 individuals representing the three target species were gathered in the western parts of the Alps and in Corsica (Supporting information Appendix S1; Supporting Data 1). Ninety‐five samples were issued from museum collections, the remaining 254 ones consisted of wing punch biopsies from individuals captured in the field. Samples originated from France (*n* = 90), Germany (*n* = 1), Italia (*n* = 27), and Switzerland (*n* = 231) and included bats caught in maternity roosts, in hunting grounds, or in transit zones. Only adult bats were genotyped in order to minimize autocorrelation between samples that could arise from mother and pup pairs. All tissues were stored in ethanol at −20°C. Genomic DNA was isolated with the DNeasy Blood & Tissue Kit (Qiagen, Switzerland). As a preliminary taxonomic assignation, individuals were sorted according to their mtDNA lineages, using an array of methods based on short fragments of the 16S gene, as described in Andriollo and Ruedi (2018). This initial mtDNA assignment resulted in 152 samples carrying mitochondrial haplotypes typical of *P. auritus*, 79 of *P. austriacus,* and 118 of *P. macrobullaris* (Supporting Data 1).

### 2.2 Amplification and genotyping of nuclear markers

Individual genotypes were further characterized by 23 autosomal microsatellite loci developed by Burland, Barratt, and Racey (1998) and Razgour et al. (2013). Five multiplex PCR amplifications (Supporting information Appendix S2) were optimized to be performed in a 10‐μl reaction volume containing 2–10 ng of genomic DNA, 5 μl HotStarTaq Master Mix, 0.3 μM of forward and reverse primers each, and completed with double distilled water. We used the following cycling protocol on a TC‐412 Programmable Thermal Controller (Techne): 35 cycles with 94°C for 30 s, 56°C for 90 s, and 72°C for 60 s. Before the first cycle, a prolonged denaturation step (95°C for 15 min) was included and the last cycle was followed by a 30‐min extension at 72°C. Amplicons were sized through fragment analysis by a commercial company (Ecogenics, Switzerland) on an ABI 3730 DNA analyser (Applied BioSystems). Alleles were then scored semi‐automatically using the microsatellite plugin in Geneious R10 (Kearse et al., 2012). Twenty‐eight individuals (8% of the dataset) were amplified and genotyped independently twice for consistency checks, but no discrepancies were observed in scoring these duplicates. We used the package poppr (Kamvar, Tabima, & Grünwald, 2014) in the R environment (R Core Team, 2017) to (a) discard individuals with more than 25% missing genotypes, (b) discard loci with more than 40% missing data in each intraspecific subset, (c) test for Hardy‐Weinberg equilibrium (hereafter HWE), and (d) calculate genetic diversity indices. Since our sampling was geographically uneven, some isolated individuals could not be aggregated into biologically meaningful populations for HWE tests. These statistical tests were thus restricted to the better sampled populations, and loci with consistent departure across these populations (i.e., showing sign of null alleles) were discarded from further analysis. The software coancestry (Wang, 2011) was used to estimate the coefficients of identity Δ_7_ and Δ_8_ described by Jacquard (1972) through the triadic likelihood method (Wang, 2007). These inbreeding coefficients can identify putative monozygotic twins (Δ_7_ = 1; Δ_8_ = 0) or parent–offspring pairs (Δ_7_ = 0; Δ_8_ = 1) that could bias allelic frequency estimations and cause departure from expectations inherent to population genetics models (Wang, 2011).

### 2.3 Interspecific analyses

In order to quantify levels of interspecific admixture, we analyzed the overall dataset with structure v. 2.3.4 (Pritchard, Stephens, & Donnelly, 2000). This Bayesian clustering method uses a MCMC algorithm to assign individuals in a preset number of groups (K) by minimizing departure from HWE and linkage disequilibrium (LD), with no prior about individual group‐membership. Ten independent chains, consisting of 100,000 generations after 50,000 generations of burn‐in, were run for each *K* ranging from 1 to 10. A model with independent allele frequencies among populations was selected. The online pipeline CLUMPAK (Kopelman, Mayzel, Jakobsson, Rosenberg, & Mayrose, 2015) was used to combine and summarize independent replicates. The most likely number of populations (*K*) was identified under the Δ*K* criterion of Evanno, Regnaut, and Goudet (2005).

The assumptions of population equilibrium models (HWE and LD) on which Bayesian clustering approaches rely (Pritchard et al., 2000) are often violated with uneven sample sizes and may bias assignments (Puechmaille, 2016). Since uneven sampling prevailed in our study, we also employed multivariate analyses that are free from these assumptions to describe the diversity of individual genotypes. These methods also perform better in detecting genetic clines as they are not forcing individual assignment into discrete clusters (Patterson, Price, & Reich, 2006). A principal component analysis (PCA) implemented in adegenet (Jombart, 2008) was carried out on the scaled allele frequencies, with missing data (<6% of genotypes) replaced by mean allele frequencies. When applicable, the elbow criterion (Ketchen & Shook, 1996) was used on the scree plot of eigenvalues to characterize the number of principal components best explaining the structure of the data.

### 2.4 Detection of hybrids

In order to estimate the detection power of interspecific hybrids provided by our multilocus genotyping approach, we applied maximum‐likelihood methods to estimate the ancestry index *S* (the amount of genetic information inherited from a given parent) and the interclass heterozygosity index *H_I_* (the proportion of loci with one allele of each parental species) on the basis of prior estimates of parental allele frequencies using the R package HIest (Fitzpatrick, 2012). We calculated parental allele frequencies on the basis of individuals classified in the interspecific structure analysis with *Q*‐values higher than 0.96, which were considered to represent pure parental individuals. Hybrid genotypes were then generated in silico from these parental pools by simulating random interspecific mating using the package adegenet. *S* and *H_I_* were estimated a posteriori for parental individuals, for simulated offspring and for individuals with uncertain parentage (i.e., those with *Q‐*values < 0.96, which were not attributed to parental populations). Since this descriptive method does not try to sort individuals into predefined classes, the power analysis relied on critical evaluation of *S* and *H_I_* values overlap between different hybrid categories.

### 2.5 Intraspecific analyses

We ran structure analyses for the three intraspecific datasets as described above, but using the correlated model for allele frequencies. Since the second‐order statistics Δ*K* cannot correctly identify the best *K* when *K* = 1, the Evanno's criterion was used in combination with the maximal logarithm probability of the data when no structure was suspected. PCA were also performed for each species independently. Intraspecific analyses of molecular variance (AMOVA) were performed with arlequin, version 3.5.2.2 (Excoffier & Lischer, 2010). In order to allow comparisons of diversity indices between the three species, only the 15 loci that consistently amplified in all of them were considered. Individuals were grouped into populations according to their geographic locations. Pairwise *F*‐statistics were calculated as the number of different alleles between microsatellite genotypes, and statistical significance evaluated using a nonparametric test with 10,000 permutations. 95% confidence intervals for fixation indices were computed with 10,000 bootstraps using the R package hierfstat (Goudet, 2005). Isolation‐by‐distance (IBD) was tested with a Mantel test (*n* = 9999 iterations), conducted between the pairwise genetic distance matrix and the matrix of Euclidian geographical distances between individuals, as implemented in adegenet (Jombart, 2008). Pairwise genetic distances were estimated as the Euclidean Reynolds’ distance (Re) (Reynolds, Weir, & Cockerham, 1983), while geographic distances were calculated with the R package geosphere (Hijmans, Williams, & Vennes, 2016), as orthodromic distances (in km) between geographical coordinates.

## 3 results

### 3.1 Multilocus genotype datasets

A minimal number of 15 loci that were reliably amplifying in all three species were kept for the interspecific dataset. For the intraspecific datasets, we retained 15 to 19 loci depending on the species. Among the discarded loci, three (Paus03, Paus11, and Paus19) could not be reliably called in all three species because of amplification artifacts. Likewise, loci Paus01, Paus07, and Paus09 in *P. auritus*, Paur06 in *P. austriacus* and Paus04 and Paus07 in *P. macrobullaris* did not amplify well in those species. Finally, two loci in *P. auritus* (Paus13 and Paus16) and a single one in *P. macrobullaris* (Paus15) showed significant and consistent departure from HWE for several populations likely due to the presence of null alleles, and were thus also discarded for these species. Other signs of disequilibrium concerned only one or two populations per species and other loci were thus kept for intraspecific analyses (Supporting information Appendix S3). Inbreeding analyses carried with coancestry did not identify twins in the dataset (all estimated Δ_7_ lower than 0.56). Moreover, the individuals exhibiting the highest kinship scores originated from different colonies or regions and were thus unlikely mother–pup pairs. Only two possible pairs of individuals having parent–offspring characteristics (Δ_7_ = 0; Δ_8_ > 0.96) were identified, but one concerned two lactating females of *P. auritus* sampled in the same breeding colony in Isère, and the other were two *P. austriacus* from distinct collection sites sampled at five years interval. Again they could likely represent related animals issued from successive years of reproduction, but since these cases were marginal, all the 349 individuals were kept in the dataset for subsequent analyses. The datasets overall contained less than 6% missing data, the mean number of alleles per locus ranged from 7.9 to 17.3 across species, with a mean heterozygosity ranging from 0.5 to 0.8 (Supporting information Appendix S4). The complete multilocus genotype dataset for the 349 individuals is provided in Supporting Data 2.

### 3.2 Interspecific analyses

**Figure 1:**
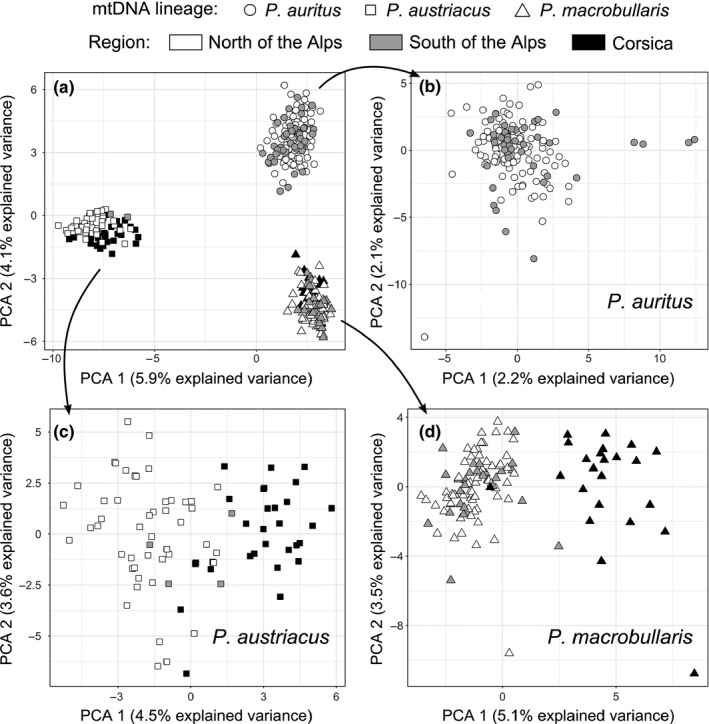
Principal component analyses based on allele frequencies of 349 long‐eared bats. Individuals are represented by different symbols according to their mitochondrial lineage and colored according to their geographical origin. PCA analyses were conducted on: (a) the interspecific dataset (308 alleles, 15 loci); (b) the intraspecific dataset for *P. auritus* (259 alleles, 15 loci); (c) the intraspecific dataset for *P. austriacus* (158 alleles, 19 loci); (d) the intraspecific dataset for *P. macrobullaris* (133 alleles, 17 loci)

For the global dataset, the Evanno's criterion calculated from Bayesian clustering analyses based on 15 loci indicated *K* = 3 groups was the most likely structure (Supporting information Appendix S5A). Clustering of individuals into these three groups was fully concordant with the initial species assignation based on mitochondrial identifications, and corresponded, respectively, to 152 *P. auritus*, 79 *P. austriacus* and 118 *P. macrobullaris*. No sign of recent admixture was detected as all but one Q‐values were greater than 0.96. This was notably the case for all *P. auritus* and *P. macrobullaris* individuals originating from breeding colonies located in the same building in Valais, or for other samples taken in close geographic proximity. A single individual sampled in Piedmont (Italy) was assigned to the *P. auritus* cluster with a slightly lower *Q*‐value (0.89), and to *P. macrobullaris* with a Q‐value of 0.11. Increasing the *K* value did not reveal any additional meaningful cluster, but rather assigned isolated individuals to a mixture of clusters (Supporting information Appendix S6). Thus, a posteriori nuclear‐based assignations to the three groups and initial mtDNA lineage identifications showed no cytonuclear discordance (Supporting information Appendix S6). The multivariate analysis indicated that the two first axes of the PCA carried about 10% of cumulated variance and represented the main structure of the dataset. The first axis perfectly discriminated all *P. austriacus* individuals from the others, while the second axis discriminated all *P. auritus* from *P. macrobullaris*, with no individual set in a genetically intermediate position (Figure 1a).

**Figure 2:**
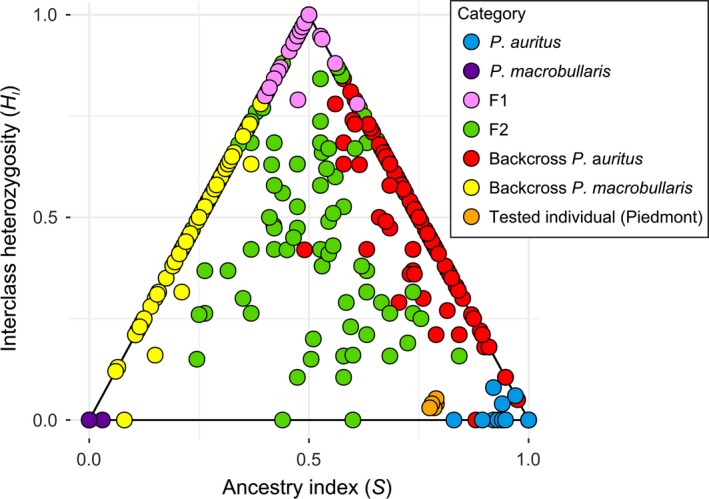
Estimates of ancestry index (*S*) and interclass heterozygosity (*H_I_*) for pure *P. auritus* and *P. macrobullaris*, for different simulated hybrid classes (F1, F2 and backcrosses) and for the problematic individual from Piedmont. Hybrids were simulated in silico by random mating of existing genotypes. The assignation of putative hybrid from Piedmont (bootstrapped 200 times) is shown in orange

Since no sign of admixture or introgression was detected in *P. austriacus* with any of the methods used, power analyses of hybrid detection were only performed for the other two, most closely related species, namely *P. auritus* and *P. macrobullaris*. All estimates of *S* in pure parental forms were either very close to 0 or to 1, whereas *H_I_* was close to zero in all of these individuals (Figure 2). For 300 simulated F1 offspring individuals, *S* and *H_I_* ranged from 0.34 to 0.58 and from 0.68 to 1, respectively. There was no overlap between values observed for parental and simulated F1 individuals, providing 100% detection power in these two categories. Furthermore, only one of 300 simulated F2 (0.3%) had *S* estimates comprised within the range of that of parental species, indicating the high detectability of these second generation hybrids given the array of 15 markers used. Other categories of hybrids (backcrosses, but also hybrids of later genealogical classes not shown) had *H_I_* estimates ranging from 0 to 1, and their *S* values overlapped rarely with that of the parental populations; hence, more than 97% of simulated F2 hybrids were unambiguously detected by this method.

The single individual from Piedmont assigned to *P. auritus* with a lower Q‐value (0.89) exhibited missing data for one of the 15 retained loci. In order to test whether this missing locus could influence its slightly lower assignation score, we employed a bootstrap approach to replace the missing data with alleles sampled according to their frequency in the parental *P. auritus* population. 200 replicates were generated and the resulting hybridization indices estimated. The positioning of this individual was consistent across all bootstrapped replicates (Figure 2), which resulted in very low *H_I_* values (0.02 to 0.05), consistent with parental *P. auritus* genotypes, but *S* values ranged from 0.78 to 0.80, falling outside values observed for parental *P. auritus* genotypes (Figure 2). The Piedmont individual did not include private alleles typical of *P. macrobullaris* in its genotype, but exhibited four alleles from several loci (allele 148 at Paus06, allele 264 at Paus05, two copies of the allele 112 at locus Paus10 and allele 137 at Paus14) that occurred at relatively low frequency in *P. auritus* (1.3%, 2.2%, 3.8% and 6.5%, respectively) while they were more common in *P. macrobullaris* (33.3%, 30.2%, 27.2% and 98.1%).

### 3.3 Intraspecific analyses

For *P. auritus*, the highest Δ*K* was observed for *K* = 6 (Supporting information Appendix S5B), although the constant decreasing of likelihood with increasing values of K and the complete admixture of individual genotypes observed in structure results for *K* > 1 (Supporting information Appendix S7) indicated no consistent substructure in this dataset. The PCA analysis confirmed that the dataset was poorly structured with some lone individuals appearing as outliers (Figure 1b) but no apparent subgroups. The axis 1 segregated four specimens originating from a colony of Losone (Ticino), while eight other genotyped individuals from this colony grouped within other *P. auritus* individuals. Population differentiation was extremely weak, although statistically significant, with fixation indices between populations (*F*_ST_) comprised between 0 and 0.02 (Supporting information Appendix S8). Inbreeding coefficients (F_IS_) showed the highest values of all three species in *P. auritus* and were significantly higher than zero for most populations (Table 1).

**Table 1:** Genetic diversity indices calculated from 15 microsatellite loci for the 22 populations sampled

Species	Region	*n*	*A*	*H* *_O_*	*H* *_E_*	*F*_IS_ [95% CI]	*P*
*P. auritus*	Isère	16	8.5 ± 2.1	0.79	0.83	0.02 [−0.04–0.15]	n.s.
	Geneva and Ain	28	10.9 ± 3.2	0.73	0.82	0.09 [0.03–0.23]	***
	Vaud	18	9.3 ± 2.9	0.76	0.80	0.02 [−0.02–0.16]	n.s.
	Valais	30	9.6 ± 3.4	0.67	0.77	0.07 [0.03–0.26]	**
	Northern Switzerland	18	9.1 ± 3.1	0.70	0.79	0.05 [0.00–0.25]	*
	Ticino and Graubünden	28	10.3 ± 2.9	0.75	0.82	0.05 [0.00–0.20]	*
	Northern Italy	8	7.3 ± 2.6	0.68	0.81	0.17 [0.07–0.27]	***
	Abruzzo and Campania	6	5.1 ± 1.3	0.63	0.75	0.13 [0.04–0.30]	**
*P. austriacus*	Corse‐du‐Sud	12	6.7 ± 2.5	0.70	0.76	0.08 [0.01–0.15]	*
	Haute‐Corse	18	6.2 ± 2.5	0.75	0.73	−0.04 [−0.10–0.03]	n.s.
	Italy	2	2.6 ± 0.8	0.73	0.74	−0.03 [−0.57–0.21]	n.s.
	Geneva and Ain	27	6.9 ± 2.7	0.71	0.72	0.00 [−0.02–0.06]	n.s.
	Vaud and Valais	11	5.7 ± 2.1	0.75	0.74	−0.01 [−0.08–0.05]	n.s.
	Northern Switzerland	9	4.9 ± 1.8	0.72	0.68	−0.08 [−0.15–0.03]	n.s.
*P. macrobullaris*	Corse‐du‐Sud	10	5.6 ± 1.8	0.64	0.73	0.11 [0.02–0.24]	n.s.
	Haute‐Corse	14	5.1 ± 2.0	0.62	0.63	−0.01 [−0.16–0.21]	n.s.
	Northern Italy	12	5.3 ± 1.8	0.59	0.67	0.07 [−0.01–0.30]	n.s.
	Isère	16	5.4 ± 1.5	0.63	0.65	0.01 [−0.14–0.26]	n.s.
	Geneva	14	5.0 ± 1.6	0.66	0.63	−0.09 [−0.14–0.08]	n.s.
	Valais	35	5.8 ± 2.2	0.57	0.63	0.07 [0.04–0.19]	*
	Ticino	17	5.8 ± 2.2	0.65	0.67	−0.04 [−0.11–0.16]	n.s.

Notes

n.s.: not significant.

Number of individuals (*n*), number of alleles per locus (A) with standard deviation, observed (H_O_) and expected (H_E_) heterozygosity, inbreeding coefficient (*F*_IS_) with 95% confidence interval and the *p*‐value of the associated permutation test (P) are provided for each population.

* *p* < 0.05,

** *p* < 0.01,

*** *p* < 0.001.

By contrast, Bayesian clustering analyses on *P. austriacus* individuals revealed a clear intraspecific subdivision, with K = 4 being identified as the most likely structure (Supporting information Appendix S5C). A first cluster mainly concerned bats from Central Europe, as opposed to Corsica and Italy (Supporting information Appendix S9). The second cluster was predominantly Corsican and Italian, while the third cluster mostly concerned five individuals sampled in the same breeding colony of Hermance (Geneva), and the fourth cluster was represented by a mixture of individuals of varied geographical locations. In the PCA, individuals from Corsica and Italy were largely discriminated from other continental samples along the first axis (Figure 1c). The AMOVA supported two distinct genetic clusters, the first grouping including individuals from Switzerland, neighboring France and Germany, and the second clustering samples from Corsica and Italy. The differentiation between populations of a same cluster was poor (pairwise *F*_ST_ lower than 0.03), while the Swiss populations were differentiated from the Italo‐Corsican group by *F*_ST_ up to 0.14 (Supporting information Appendix S8). This differentiation was not likely explained by inbreeding of populations, as all 95% CI of *F*_IS_ comprised zero and the single statistically significant *F*_IS_ (population from Corse‐du‐Sud) was weak (Table 1)*.*

Bayesian clustering analysis showed a clear population differentiation with *K* = 2 groups in *P. macrobullaris* (Supporting information Appendix S5D). The two clusters were largely congruent with the geographic origin of individuals, the first one including individuals from mainland France, Switzerland, and Italy, and the second one comprising bats from Corsica (Supporting information Appendix S10). One exception was a single individual from Corsica that was strongly assigned to the mainland cluster, and few individuals from Northern Italy and one from Valais exhibiting intermediate assignation (Supporting information Appendix S10). The PCA analysis supported the same subdivision as structure (Figure 1d). The AMOVA also recovered two main genetic clusters corresponding to Corsica and mainland. The intra‐group fixation indices were all very low (pairwise *F*_ST_ lower than 0.03), except for the Geneva population which was distinct from Isère and Valais (*F*_ST_ = 0.07; Supporting information Appendix S8). This differentiation of the Geneva population was not explained by inbreeding artifacts: its *F*_IS_ was not statistically significant indicating the relatedness of individuals was not different from that expected under a model of random mating (Table 1). The single statistically significant *F*_IS_ (population from Ticino) was about 0.07.

**Figure 3:**
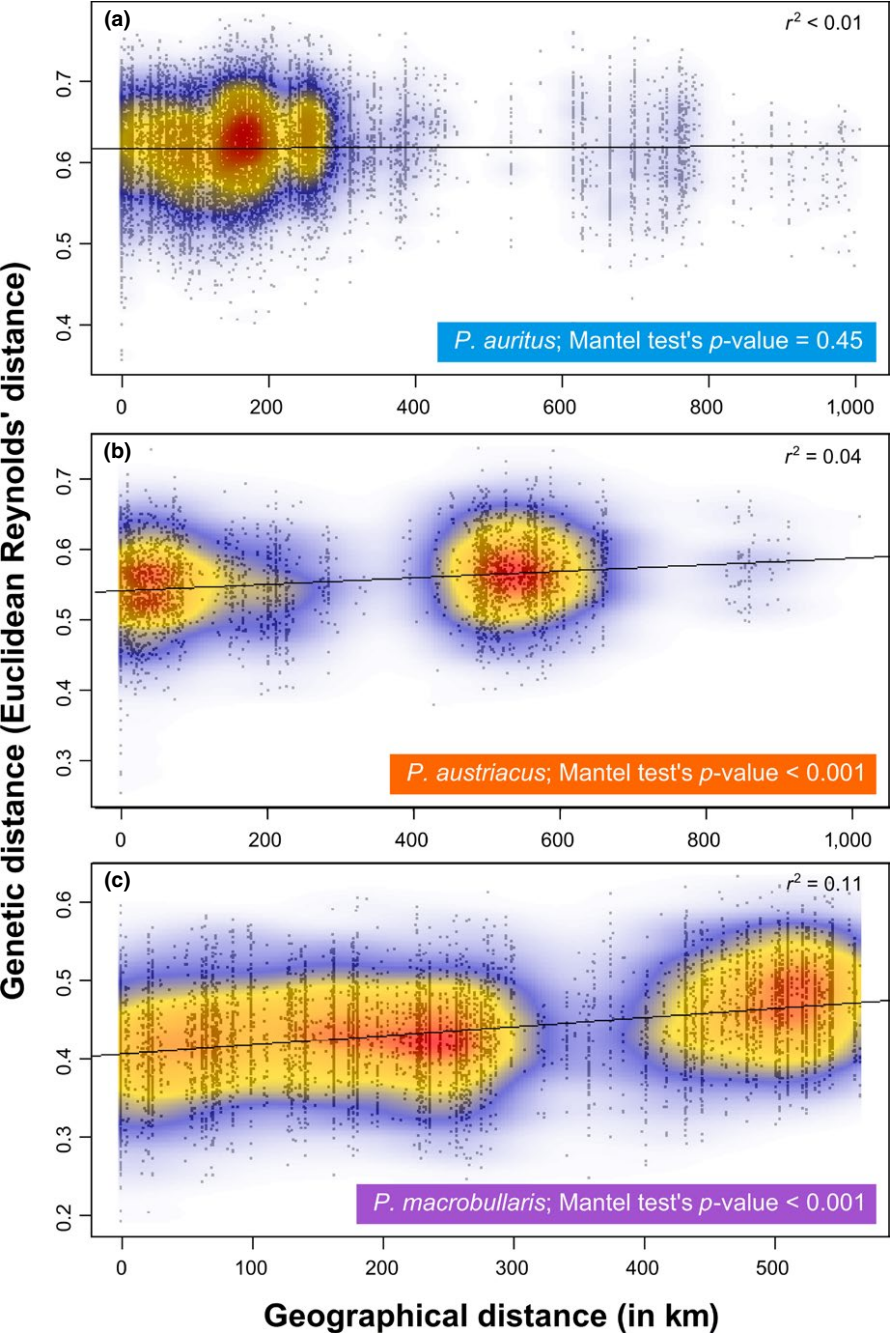
Bivariate plot of the relationship between genetic and geographical distances for the three species datasets used: (a) *P. auritus*, (b) *P. austriacus,* and (c) *P. macrobullaris*. The black line represents the linear curve best fitting the data. For readability purposes, a two‐dimensional kernel density estimation heat map is superimposed to the scatterplot, with red for highest densities

Test of IBD pattern among individuals from each of the three species (Figure 3) indicated no or weak relationship. This is most remarkable in *P. auritus*, where no significant relation was found in the dataset comprising individuals sampled over 1,000 km apart (Figure 3a). Also evident is the clusters of comparisons found in *P. austriacus* and *P. macrobullaris* near 500 km (Figure 3b and c), which reflects the numerous samples originating from Corsica or the Alps and separated by this geographical distance. These clusters of comparisons also reflect the lack of individuals from intermediate locations (Supporting information Appendix S1).

## 4 discussion

### 4.1 Complete reproductive isolation among species

Despite several situations of strict sympatry among the three sampled species, and their conservative morphology, all conducted genetic analyses indicated that reproductive barriers between those species are not porous. Indeed, no recent hybrid was detected by the nuclear markers, including between individuals from distinct species sharing the same roost. A single individual from north‐eastern Italy exhibited a slightly lower cluster assignment (*Q* = 0.89) due to the presence in this bat of otherwise uncommon alleles in *P. auritus*. Our simulation analyses suggest that this intermediate assignment is unlikely due to past introgression of alleles from *P. macrobullaris*, but might rather reflect the existence of a slightly diverging local population of *P. auritus*. More samples from this area would be required to tackle this question more precisely. Additionally to the observed absence of contemporary nuclear gene flow among species, mitochondrial identifications of all individuals perfectly matched the species assignation inferred by microsatellite analyses, as each group was characterized by typical nuclear genotypes and carried species‐specific mitochondrial lineage. This complete cytonuclear concordance indicates a lack of historical introgression between the three taxa. We thus confirm that the use of simple mitochondrial barcodes (typically COI) is perfectly suited to identify these long‐eared bat species in Western Europe despite the existence of morphologically intermediate individuals (Andriollo & Ruedi, 2018). This concordance of genetic markers and lack of interspecific gene flow also validates previous genetic assignations that were exclusively based on mitochondrial markers (Ashrafi, Beck, Rutishauser, Arlettaz, & Bontadina, 2011; Mattei‐Roesli, 2010). This clear‐cut situation of strong barriers to gene flow among the three *Plecotus* contrasts with the situation of other pairs of cryptic species such as *Myotis* bats which hybridize and exchange both nuclear and mitochondrial genes (Berthier et al., 2006; Morales & Carstens, 2018; Morales et al., 2017).

Clearly, behavioral or reproductive barriers prevent effective introgression in this species system. As their close morphological resemblance would a priori not mechanically imped mating, other mechanisms such as echolocation calls (Altringham & Fenton, 2003; Puechmaille et al., 2014; Schuchmann, Puechmaille, & Siemers, 2012), olfactory compounds (Horáček, 1975; Stebbings, 1966), species‐specific mating display or phenologies may be effective barriers to the interspecific breeding in these bats.

### 4.2 Porous geographical barriers for local populations

The Alps have been a strong barrier to gene flow for many organisms while they recolonized suitable habitats northwards after glaciations (Hewitt, 1999). Despite their potential for overtaking topographic obstacles, most species of bats tested so far recolonized Central and Northern Europe from the Southeast (Balkans) or Southwest (Iberia) but rarely from the southern Italian peninsula (the Apennines), probably due to the presence of the alpine range (Alberdi, Gilbert, et al., 2015b; Flanders et al., 2009; Petit, Excoffier, & Mayer, 1999; Razgour et al., 2013; Wright et al., 2018). Only *Barbastella barbastellus* seems to have recolonized northern parts of its range from Italy (Rebelo et al., 2012), while fairly limited transalpine migration has been noted in few species such as *M. myotis* and *M. blythii* (Castella et al., 2001). For the lowland‐adapted *P. austriacus*, Razgour et al. (2013) reported a strong differentiation of Italian versus Central and Western European populations, with directional gene flow from the Italian Peninsula toward Western Europe, following a species’ range constriction and subsequent expansion due to climate change (Razgour et al., 2017). Although a strong effect of the Alps as a blocking orographic barrier to gene flow is observed in this species (Razgour et al., 2014), limited gene flow occurs between the peninsula and more central areas, which might have resulted from dispersal around the Alpine massif, notably along the Mediterranean or Adriatic coasts, more than by crossing Alpine passes. Despite an overall low genetic structuration in this species, there was clear signal of differentiation between the Italo‐Corsican population and the other populations we investigated in other parts of continental Europe. Although Bayesian cluster analyses (Supporting information Appendix S7) and PCA (Figure 1b) showed no complete partition of genotypes north and south of the Alps, the Italian and Corsican samples tend to be segregated from other, more northerly samples. Finally, although higher altitudes are clearly avoided by *P. austriacus* in the Alps (Andriollo & Ruedi, 2018; Rutishauser et al., 2012), the species commonly occurs in mountainous areas in Corsica, with an altitudinal distribution ranging from sea level to 2’150 m (Courtois et al., 2011). This observation suggests the ecology of the insular populations, although genetically similar to their relatives from Italy, differ from those of the mainland, and that *P. austriacus* is ecologically more plastic than previously thought.

As predicted for the high‐altitude adapted species (Alberdi et al., 2013; Alberdi, Garin, Aizpurua, & Aihartza, 2012), *P. macrobullaris* does not show any substructure between populations sampled north and immediately south of the Alps. Even the population sampled at the lowest elevation, below 500 m a.s.l., in Geneva and Ticino, at either side of the Alps, do not exhibit significant differentiation (Supporting information Appendix S10 and Figure 1d). It seems that *P. macrobullaris* potentially occupies the habitats found across its considerable elevation range (Alberdi et al., 2013; Andriollo & Ruedi, 2018; Rutishauser et al., 2012), which enables good connectivity of populations in the Alps. Within Corsica too, this species is commonly found from sea level to the highest parts of the island (Courtois et al., 2011; pers. obs.), which again would facilitate exchanges across potential orographic barriers.

Finally, the more ubiquitous and altitudinally broadly distributed species *P. auritus* also shows a minimal effect of the alpine barrier on its population structure, as all our genetic analyses do not support any significant subdivision of populations (Figure 1b) at this geographical scale. The Alpine range is thus a very porous barrier at least for both *P. macrobullaris* and *P. auritus*. Consistent with this negligible effect of orographic barriers in these species, major swarming areas and high‐altitude mountain passes located well above 1,500 m a.s.l. are used by numerous *P. auritus* during the autumnal mating period (Aellen, 1961; Groupe d'études faunistiques de Jaman, 2017; Patthey, 2014; Ruedi et al., 1989). Clearly these areas provide mating opportunities for individuals issued from vast catchment areas (Rivers, Butlin, & Altringham, 2006), and thus potentially connects populations with high gene flow even across the Alps, as suggested by our analyses. However, the mating behavior is largely unknown in *P. macrobullaris*, and further knowledge is required to better understand factors promoting gene flow in this species.

Regarding the effect of open sea as a barrier to gene flow for long‐eared bats, our predictions arising from the patterns of island occupation in the Mediterranean is also largely corroborated by the results of nuclear genetic structure analyses. Except for *P. auritus*, which apparently does not occur on Corsica (Courtois et al., 2011), the effect of the Ligurian Sea is minimal for the potentially good colonizing species (*P. austriacus*), but more significant for the highland‐adapted, apparently poor colonizer *P. macrobullaris*. Indeed, the few individuals of *P. austriacus* from mainland Italy appear to be genetically identical to animals from Corsica and differ from those sampled elsewhere in continental Europe (Supporting information Appendix S9 and Figure 1c). This differentiation, however, also coincides with the Alpine range and might in part be due to an isolation‐by‐distance effect. As *P. austriacus* is very rare or absent from the intervening, northern regions of Italy immediately adjacent to the Alps, a proper sampling design to test these effects seems difficult to implement in the field. Clearly, populations sampled in south‐eastern France, that is, in the area bordering the Ligurian Sea along the occidental limits of the Alps, could bring useful insights on gene flow and migration routes. Elsewhere, in the Italian Peninsula and Sardinia, no study involving nuclear variation have been conducted so far, but the limited information issued from mitochondrial lineages (Galimberti et al., 2012; Razgour et al., 2013) suggests that *P. austriacus* from this region are not particularly differentiated.

Regarding the effect of open sea barriers, an opposite situation prevails for *P. macrobullaris*, as all individuals from Corsica form a distinct cluster relative to those from the continent (Supporting information Appendix S10 and Figure 1d). The only exception is an animal from Porto‐Vecchio in south‐eastern Corsica, which exhibits a multilocus genotype identical to that of continental samples. This exceptional similarity is not due to an analytical error, as its genotype was extracted and characterized twice independently without visible inconsistency. This animal might therefore represent a recent migrant from the continent, indicating that the Ligurian Sea is not an absolute barrier for *P. macrobullaris*, although such occasional gene flow is not sufficient to counter the local differentiation of Corsican populations.

### 4.3 Implications for conservation

In P. auritus, phylogeographic studies based on mitochondrial markers showed the species was highly structured across its entire range (Bogdanowicz et al., 2015; Çoraman et al., 2013; Galimberti et al., 2012; Ibáñez, García‐Mudarra, Ruedi, Stadelmann, & Juste, 2006; Juste et al., 2004; Kiefer, Mayer, Kosuch, Von Helversen, & Veith, 2002; Kruskop, Borisenko, Ivanova, Lim, & Eger, 2012; Pestano, Brown, Suarez, Benzal, & Fajardo, 2003; Spitzenberger et al., 2006; Veith, Beer, Kiefer, Johannesen, & Seitz, 2004). In particular, two divergent lineages distributed in Central Europe and mainland Italy (“Western” and “Eastern” lineages in Supporting information Appendix S11) were considered to potentially deserve sub‐specific division (Spitzenberger et al., 2006), and a third one (the “Abruzzo lineage” in Supporting information Appendix S11) was even qualified as an “unconfirmed candidate species” (UCS) by Galimberti et al. (2012). Our analyses of nuclear DNA show no genetic breaks between populations bearing these lineages, suggesting that individuals are interbreeding regardless of their mtDNA lineage. These mtDNA variants are better considered as deep conspecific lineages (Padial, Miralles, Riva, & Vences, 2010), which mirror the situation found in other bats from this region (Andriollo, Naciri, & Ruedi, 2015). Likewise, without evidence from biparentally inherited markers, it seems premature to consider the Iberian lineage associated to the taxon *begognae* (Ibáñez, et al., 2006) as specifically distinct on the sole basis of mitochondrial data (Mayer, Dietz, & Kiefer, 2007). The apparent contrast between strong population structure inferred from mitochondrial markers versus weak differentiation inferred with nuclear markers is not uncommon in bats and may be a consequence of a stronger female philopatry compared to male migration/dispersal (Burland & Worthington‐Wilmer, 2001). Long‐eared bats fit this pattern, as shown in *P. auritus*, where the strong philopatry of females (which transmit the mitochondrial lineages to the next generations) evidenced in the breeding colonies is counterbalanced by extensive male‐mediated gene flow (Burland, Barratt, Beaumont, & Racey, 1999; Burland, Barratt, Nichols, & Racey, 2001). This increased male‐mediated gene flow is particularly evident at the autumn and spring swarming sites, where females from distant colonies may mate with unrelated males (Furmankiewicz & Altringham, 2007).

The other two species of long‐eared bats, *P. austriacus* and *P. macrobullaris*, do not exhibit any major phylogeographic pattern at mtDNA markers in Central Europe, although major distinct lineages exist in Iberia for *P. austriacus* (Razgour et al., 2013) and in the eastern Balkans for *P. macrobullaris* (Alberdi, Gilbert, et al., 2015b). However, despite the considerable mitochondrial uniformity exhibited by both species, our nuclear DNA analyses showed they were each subdivided at least into two distinct genetic pools, each corresponding to particular geographic entities (Corsica and/or Italy vs. Central Europe). Furthermore, the broader altitudinal range occupied by both species in Corsica (Courtois et al., 2011) might either be due to the milder climate found in this area, or suggests that these distinct gene pools underlie important local adaptations. In both cases and in order to preserve distinct potentials for evolution, each of these gene pools should be considered as evolutionarily significant units (Moritz, 1994), although again no mitochondrial differentiation was observed at this geographic scale (Kiefer, 2007).

## 5 conclusions

Our genetic analyses, combining mitochondrial and nuclear markers, showed no evidence of interspecific admixture between the three long‐eared bat species in the Alpine and adjacent areas, where their distributions overlap extensively. These cryptic species therefore mate assortatively based on other clues than external morphology. Furthermore, despite striking phenotypic similarities, species‐specific responses to topographic barriers could be evidenced, which correlated to distinct local altitudinal preferences of the species. The Alps, which act as an orographic barrier to gene flow in several European bats, were surprisingly porous for two of the long‐eared bat species studied (*P. auritus* and *P. macrobullaris*), but more effective in the third (*P. austriacus*). Similarly, the 100‐km‐wide channel of the Ligurian Sea also appeared to be a variably porous barrier for the two species sampled in Corsica (*P. austriacus* and *P. macrobullaris*). Overall, at the geographic scale envisioned here, these allegedly poor dispersers seem to cope well with these topographic barriers, but to different degrees. These differences led to idiosyncratic phylogeographic histories in the three species, resulting in distinct contemporary genetic structuring of populations. These intraspecific structures inferred from nuclear data largely contradict those suggested by previous analyses based on mitochondrial markers. Although three major mtDNA lineages were documented in *P. auritus*, all belong to a single nuclear genetic pool and should therefore be managed as a single unit of conservation. Conversely, a single mitochondrial lineage characterized each of *P. austriacus* and *P. macrobullaris*, yet microsatellites data segregated two well‐defined ESUs within both these species. This highlights again the importance of defining priority conservation units on the basis of multiple markers. Simple barcode approaches are certainly useful for inferring phylogeographic patterns, but not particularly suited for estimating levels of gene flow among lineages.

## ethical statement

Most tissue samples were issued from museum collections or from biopsy samples (3 mm in diameter) achieved from monitoring programs. Additional biopsy samples specifically collected for this study were performed under appropriate licenses and ethical approval (“autorizzazione cantonale TI‐05–2016” in Ticino, Switzerland; “Programme Régional de Conservation des Chiroptères en Corse” in Corsica, France; “arrêté préfectoral n^o^ 38–2016–06–28–005” in Isère, France). Capture methods included mist nets, harp traps, and hand‐net (Kunz & Kurta, 1988; Kunz, Hodgkison, & Weise, 2009). Animals were retained for a maximum of 20 min in cotton bags, before being released on the spot of capture.

## CONFLICT OF INTEREST

None declared.

## author contributions

MR TA conceived and designed the experiments and wrote the first draft. MR TA RA SA conducted the sampling. TA performed the laboratory work and computational analyses. TA MR RA contributed to the final manuscript.

## data accessibility

Supporting Information: Appendices. Zenodo doi:10.5281/zenodo.1485040. Supporting Data 1: Sampling information. Zenodo doi:10.5281/zenodo.1482057. Supporting Data 2: Multilocus microsatellite genotypes. Zenodo doi:10.5281/zenodo.1482057.
