# Supplementary material for: Porous barriers? Assessment of gene flow within and among sympatric long‐eared bat species
Source: Ecol Evol. 2018 Dec 7;8(24):12841–54. doi: 10.1002/ece3.4714 (PMC6309003; doi:10.1002/ece3.4714)
Supplement: Supplementary file 1 [file ECE3-8-12841-s001.docx]

**Appendices for:**

**Porous barriers? Assessment of gene flow within and among sympatric long-eared bat species.**

Tommy Andriollo, Sohrab Ashrafi, Raphaël Arlettaz & Manuel Ruedi

Appendix 1. Map showing the sampling localities of the 349 genotyped *Plecotus* individuals in Western Alps and Corsica. Colours represent different species (blue: *P. auritus*; orange: *P. austriacus*; violet: *P. macrobullaris*). Circle size reflects the number of sampled specimens.


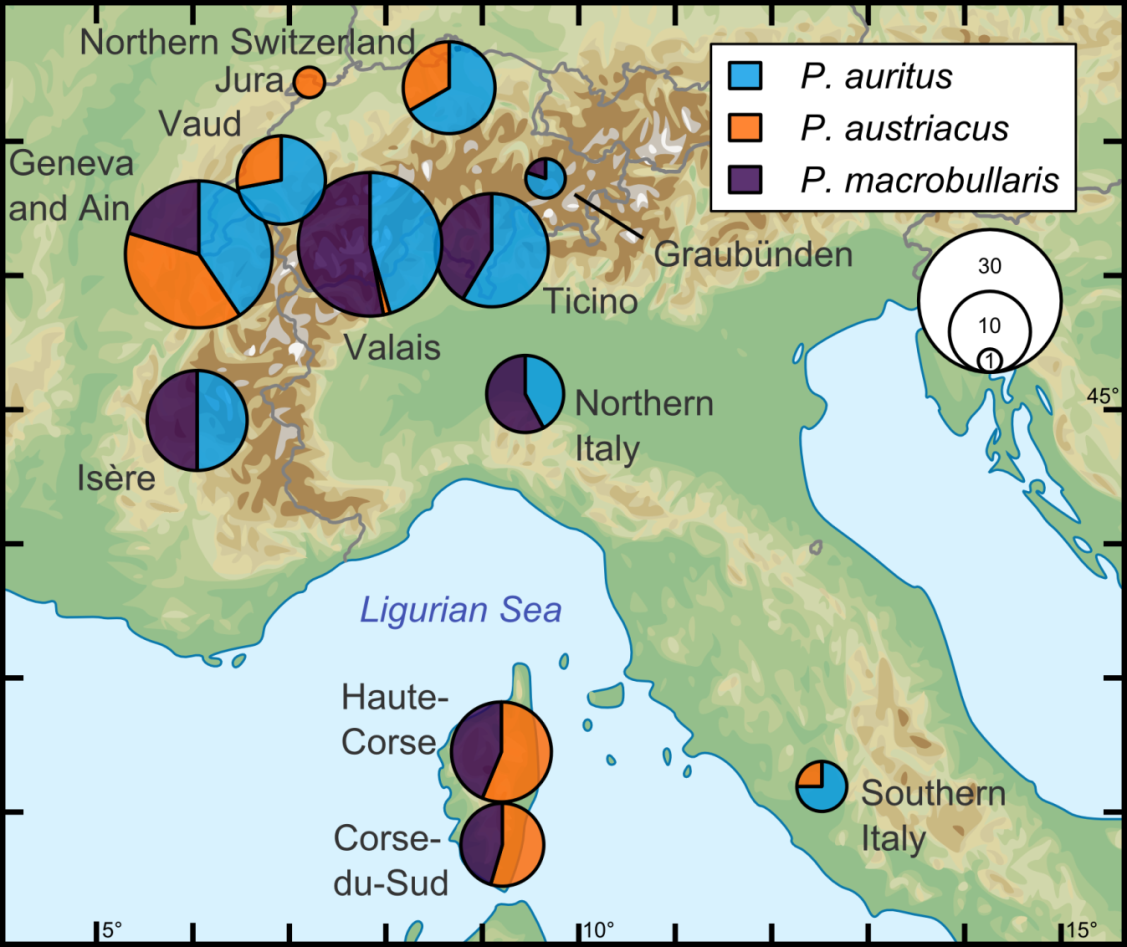


Appendix 2. Conditions used for amplification of the 23 microsatellite loci. Size range of amplicons (in base pairs) is provided for each of the three *Plecotus* species. The three italicised loci could not be reliably scored and were excluded from further analyses.

| Locus | Forward primer sequence 5’- 3’ | Reverse primer sequence 5’- 3’ | Repeat type | Size range *P. auritus* | Size range *P. austriacus* | Size range *P. macrobullaris* | Fluorescent dye | Multiplex assay |
| --- | --- | --- | --- | --- | --- | --- | --- | --- |
| Paus20 | CGGAGACTGGAGGAGTACG | AATTCGCAGCTGTCCTACC | GT | 96-120 | 108-118 | 96-104 | Atto532 | 1 |
| Paur01 | CAATTTCAAGGCAGTGCTC | TGCTGTCCCTGCATGCTG | GT | 154-174 | 144-170 | 156-172 | Atto550 | 1 |
| Paus16 | AAGTGGGACTGGAGCTGGTC | GGGTGCTTGGTGACACTGAC | CA | 138-188 | 159-188 | 152-212 | FAM | 1 |
| Paus02 | CATTTCATGGGTCCTGTTCC | TGCTAATTGACTATTCTCATCTTTGG | CA | 181-212 | 200-222 | 177-177 | Atto565 | 1 |
| *Paus03* | *GGCTTTCCAATAGAACATCCTTC* | *GGAGGCAACCAATCATCG* | *GA* | *—* | *—* | *—* | *Atto532* | *1* |
| Paus10 | CCCTCTACCCATTCAACAAGC | CGCTGATGTTACAACTCATTGTG | CA | 99-125 | 111-143 | 102-121 | Atto532 | 2 |
| Paus12 | AGCGAGCAATCAAACTCCTG | AATCAGGATGCAGCTGGAAG | GATA | 134-158 | 144-178 | 134-154 | FAM | 2 |
| Paus17 | TTGGCAGTCTCTATCCCAAG | ACAGTCAGCCAGGAAATCAC | GT | 193-211 | 185-197 | 187-203 | Atto550 | 2 |
| *Paus19* | *TGAATGCAGGCCTAACTGAC* | *CACATAAGAATCAACCAATAGATGC* | *GT* | *—* | *—* | *—* | *Atto565* | *2* |
| Paus09 | GGTGGGAATGAAACATCCAC | AAATTTGGGAGGAAGCAAGG | CA | 264-274 | 264-272 | 264-272 | FAM | 2 |
| Paus15 | GATGAAGAGTCCATGGTGTTCTG | TACCCTCGCTCTGAGGACTG | GT | 117-147 | 104-139 | 109-143 | FAM | 3 |
| Paur06 | GATCAGATTTCCAAACAGAG | AGGTTCTTTCTTCAGCTATG | (AC)(AG) | 174-236 | 156-178 | 156-222 | Atto565 | 3 |
| *Paus11* | *TCATTGCAACATTATTTATAGTAGCC* | *ACCCTCAAAGTTTATCCATG* | *CA* | *—* | *—* | *—* | *Atto550* | *3* |
| Paur05 | GGACAGTATGCCATGTTATGCTG | GCACTTTCACAAACCTAGATGG | GT | 232-256 | 238-258 | 228-244 | Atto532 | 3 |
| Paus04 | GTCCTCCCAAGCTCACCTG | ATGGAGGAGGTAACCCATCG | TTTC | 309-383 | 263-307 | 316-348 | FAM | 3 |
| Paus14 | AGTGACCAGTCATTCCAGTCG | CACTCCAGGCCTTCACTACC | GATA | 131-149 | 167-187 | 135-173 | FAM | 4 |
| Paus08 | TTGCAATGGTTCTGATGAGG | TAGCTTCCCTGTCCATCCAC | CA | 149-191 | 157-181 | 167-189 | Atto565 | 4 |
| Paus13 | TGAGCCAATACCTTATAACAAACC | GCAAGATAGAATATGGGCACTG | GATA | 189-219 | 182-208 | 186-213 | Atto550 | 4 |
| Paus07 | GGCTCCTGCACTGTGACC | GACTGCAGCCAACACGAAG | CA | 222-234 | 222-254 | 240-252 | Atto532 | 4 |
| Paus18 | TGGAATAGTTCCATGTCTTGACTC | GCAGTGCTTAACTCAGGAACAG | CA | 151-177 | 147-163 | 161-171 | Atto532 | 5 |
| Paus06 | AAGGAAGGACAACTGGAACAG | AACTGAATAGACAACTCAGATTCTCC | CA | 146-172 | 144-162 | 146-176 | FAM | 5 |
| Paus01 | TCATTGACCAAGAAATTACTAGTTAGG | AGCCAAATTGGTAGGAAACC | TATC | 208-243 | 192-212 | 192-216 | Atto550 | 5 |
| Paus05 | CCAGAGTGGGAAGTAAATCTGC | TGTTTATCAGCATGAAGGCATTAG | CA | 246-281 | 240-264 | 248-272 | Atto565 | 5 |

Appendix 3. Heat map representing *p*-values associated to HWE tests for all populations and microsatellite loci. *p*-values greater than 0.05 (HWE hypothesis not rejected) were set to 1 for representation purposes. Blank lines represent loci removed because of high levels of missing data.


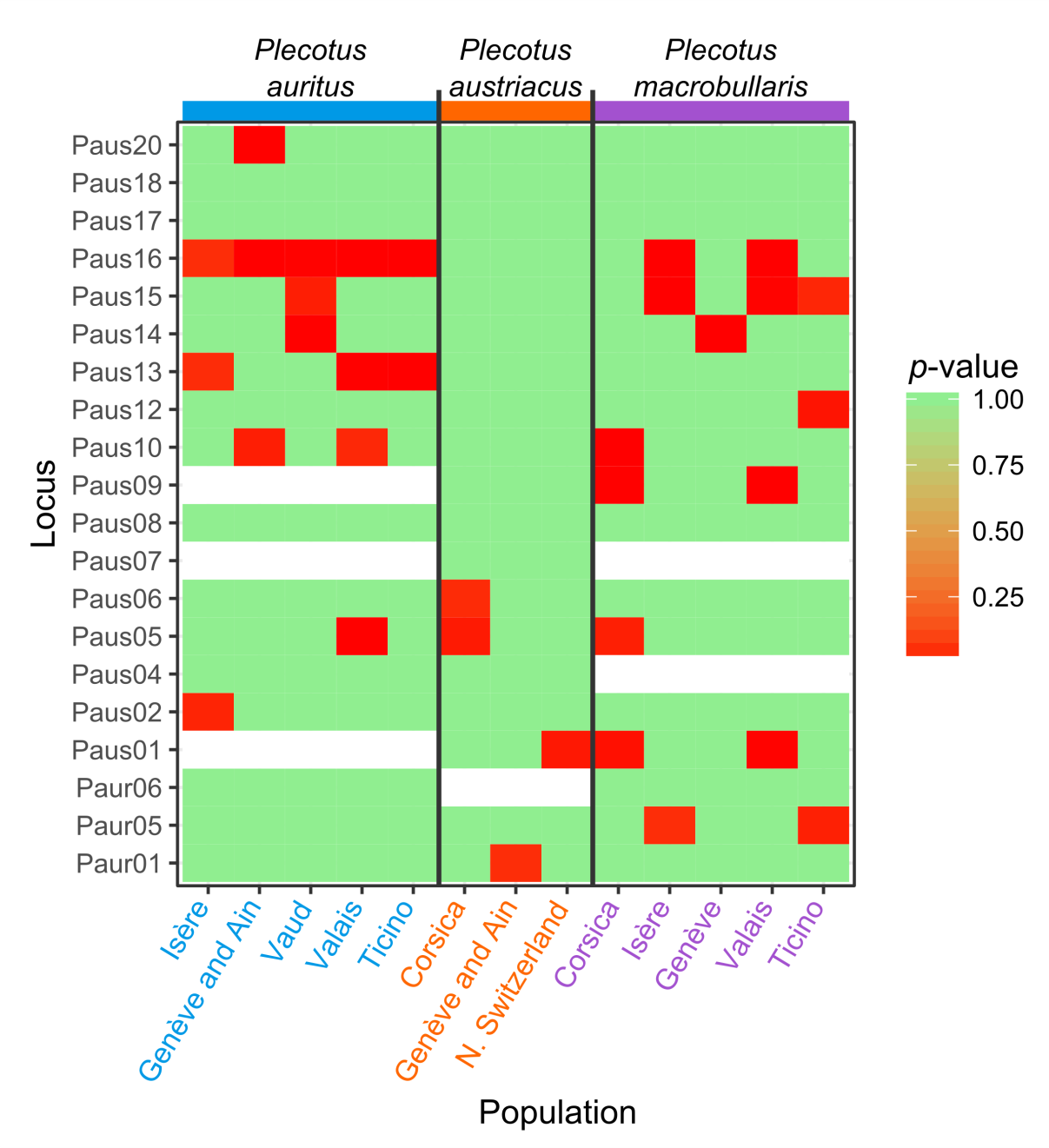


Appendix 4. Standard diversity indices of microsatellite loci retained in the four analysed genotype datasets. Number of individuals kept (n), number of loci and missing data are given for each dataset; number of alleles per locus (A), observed (H_O_) and expected (H_E_) heterozygosity are provided for each locus in each dataset, with their mean values (and standard deviation) computed for each dataset.

| Dataset | n | Number of loci | Missing data | Locus | A | H_O_ | H_E_ |
| --- | --- | --- | --- | --- | --- | --- | --- |
| Three species | **349** | **15** | **3.38%** | Paus20 | 13 | 0.37 | 0.56 |
|  |  |  |  | Paus18 | 16 | 0.73 | 0.85 |
|  |  |  |  | Paus17 | 23 | 0.78 | 0.89 |
|  |  |  |  | Paus16 | 31 | 0.43 | 0.91 |
|  |  |  |  | Paus15 | 19 | 0.71 | 0.92 |
|  |  |  |  | Paus14 | 16 | 0.39 | 0.78 |
|  |  |  |  | Paus13 | 26 | 0.73 | 0.89 |
|  |  |  |  | Paus12 | 15 | 0.71 | 0.83 |
|  |  |  |  | Paus10 | 34 | 0.62 | 0.93 |
|  |  |  |  | Paus08 | 22 | 0.69 | 0.88 |
|  |  |  |  | Paus06 | 17 | 0.80 | 0.91 |
|  |  |  |  | Paus05 | 27 | 0.78 | 0.92 |
|  |  |  |  | Paus02 | 18 | 0.55 | 0.84 |
|  |  |  |  | Paur05 | 17 | 0.75 | 0.88 |
|  |  |  |  | Paur01 | 14 | 0.83 | 0.86 |
|  |  |  |  | **Mean** | **20.5 ± 6.4** | **0.7 ± 0.2** | **0.9 ± 0.1** |
| *P. auritus* | **152** | **15** | **3.46%** | Paus20 | 13 | 0.42 | 0.45 |
|  |  |  |  | Paus18 | 14 | 0.89 | 0.89 |
|  |  |  |  | Paus17 | 19 | 0.86 | 0.89 |
|  |  |  |  | Paus15 | 16 | 0.83 | 0.91 |
|  |  |  |  | Paus14 | 6 | 0.53 | 0.58 |
|  |  |  |  | Paus12 | 7 | 0.70 | 0.74 |
|  |  |  |  | Paus10 | 24 | 0.62 | 0.94 |
|  |  |  |  | Paus08 | 22 | 0.91 | 0.93 |
|  |  |  |  | Paus06 | 13 | 0.82 | 0.87 |
|  |  |  |  | Paus05 | 23 | 0.86 | 0.92 |
|  |  |  |  | Paus04 | 38 | 0.89 | 0.94 |
|  |  |  |  | Paus02 | 11 | 0.87 | 0.86 |
|  |  |  |  | Paur06 | 28 | 0.92 | 0.94 |
|  |  |  |  | Paur05 | 15 | 0.85 | 0.86 |
|  |  |  |  | Paur01 | 10 | 0.83 | 0.81 |
|  |  |  |  | **Mean** | **17.3 ± 8.6** | **0.8 ± 0.2** | **0.8 ± 0.1** |
| *P. austriacus* | **79** | **19** | **1.33%** | Paus20 | 5 | 0.73 | 0.67 |
|  |  |  |  | Paus18 | 9 | 0.79 | 0.82 |
|  |  |  |  | Paus17 | 6 | 0.54 | 0.53 |
|  |  |  |  | Paus16 | 10 | 0.85 | 0.84 |
|  |  |  |  | Paus15 | 14 | 0.85 | 0.87 |
|  |  |  |  | Paus14 | 8 | 0.59 | 0.63 |
|  |  |  |  | Paus13 | 11 | 0.85 | 0.84 |
|  |  |  |  | Paus12 | 8 | 0.69 | 0.75 |
|  |  |  |  | Paus10 | 17 | 0.86 | 0.90 |
|  |  |  |  | Paus09 | 2 | 0.38 | 0.42 |
|  |  |  |  | Paus08 | 2 | 0.28 | 0.34 |
|  |  |  |  | Paus07 | 12 | 0.89 | 0.86 |
|  |  |  |  | Paus06 | 8 | 0.75 | 0.80 |
|  |  |  |  | Paus05 | 8 | 0.66 | 0.73 |
|  |  |  |  | Paus04 | 6 | 0.76 | 0.77 |
|  |  |  |  | Paus02 | 11 | 0.83 | 0.85 |
|  |  |  |  | Paus01 | 6 | 0.79 | 0.77 |
|  |  |  |  | Paur05 | 8 | 0.76 | 0.75 |
|  |  |  |  | Paur01 | 12 | 0.82 | 0.84 |
|  |  |  |  | **Mean** | **9.3 ± 3.6** | **0.7 ± 0.2** | **0.7 ± 0.2** |
| *P. macrobullaris* | **117** | **17** | **5.63%** | Paus20 | 4 | 0.03 | 0.04 |
|  |  |  |  | Paus18 | 6 | 0.49 | 0.54 |
|  |  |  |  | Paus17 | 9 | 0.83 | 0.81 |
|  |  |  |  | Paus16 | 5 | 0.3 | 0.59 |
|  |  |  |  | Paus14 | 5 | 0.03 | 0.04 |
|  |  |  |  | Paus13 | 13 | 0.8 | 0.81 |
|  |  |  |  | Paus12 | 6 | 0.73 | 0.72 |
|  |  |  |  | Paus10 | 11 | 0.47 | 0.71 |
|  |  |  |  | Paus09 | 4 | 0.09 | 0.14 |
|  |  |  |  | Paus08 | 8 | 0.68 | 0.68 |
|  |  |  |  | Paus06 | 12 | 0.81 | 0.81 |
|  |  |  |  | Paus05 | 11 | 0.75 | 0.8 |
|  |  |  |  | Paus02 | 1 | 0 | 0 |
|  |  |  |  | Paus01 | 9 | 0.59 | 0.82 |
|  |  |  |  | Paur06 | 18 | 0.87 | 0.88 |
|  |  |  |  | Paur05 | 3 | 0.66 | 0.6 |
|  |  |  |  | Paur01 | 9 | 0.82 | 0.78 |
|  |  |  |  | **Mean** | **7.9 ± 4.3** | **0.5 ± 0.3** | **0.6 ± 0.3** |

Appendix 5. Log likelihood and Evanno’s ΔK for Structure runs of the four datasets studied: A) the inter-specific dataset, B) *P. auritus*, C) *P. austriacus* and D) *P. macrobullaris*. The log likelihood of the data L(K) is represented by boxplots summarizing values of ten replicates for each value of K and is scaled on the left axis. ΔK is represented as hollow circles connected by dashed lines and is scaled on the right axis.


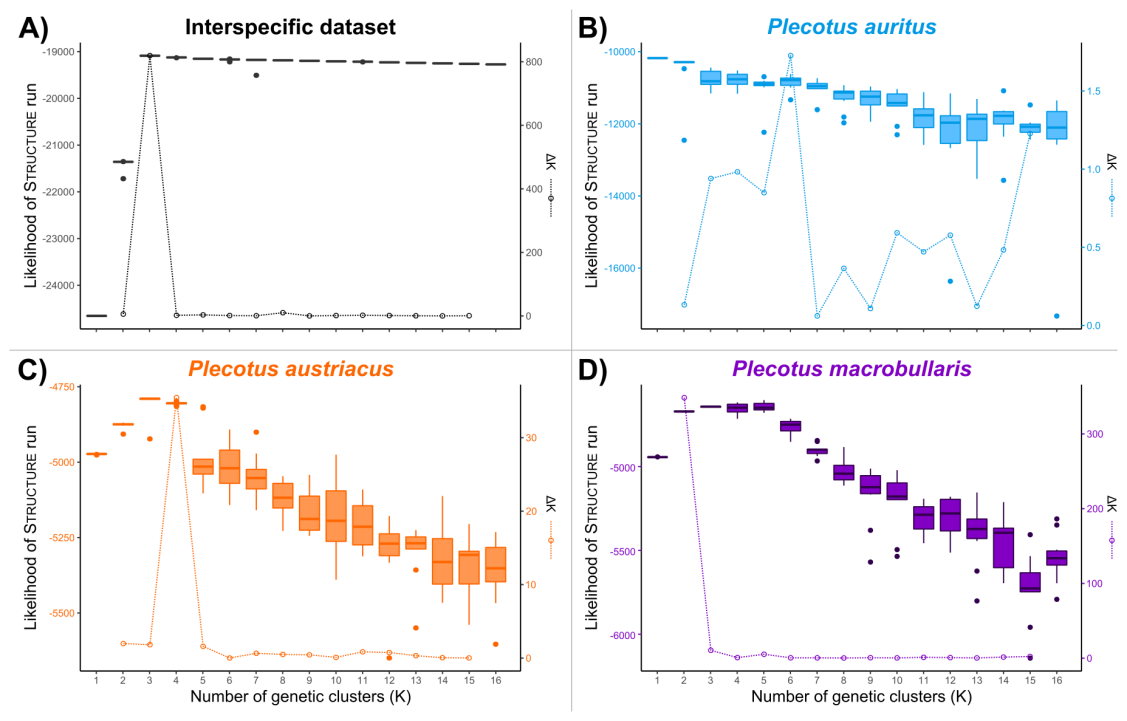


Appendix 6. Mitochondrial identification and coancestry coefficients calculated by Structure for K = 3 to 8 from the microsatellite genotypes of 349 *Plecotus* individuals. Each individual is represented by a vertical bar. On the top panel, identification based on the 16S mitochondrial gene (mtDNA) is provided. On the next six panels, coancestry coefficients estimated from nuclear genotypes (nucDNA) are represented for each individual by colour portions of different sizes. Individuals are sorted by geographical region (separated by vertical black lines). Populations abbreviations used are: GE – Geneva, GR – Graubünden, IT – Italy, NCO – Northern Corsica, NIT – Northern Italy, NSW – Northern Switzerland, SCO – Southern Corsica, SIT – Southern Italy, TI – Ticino, VD – Vaud and VS – Valais.


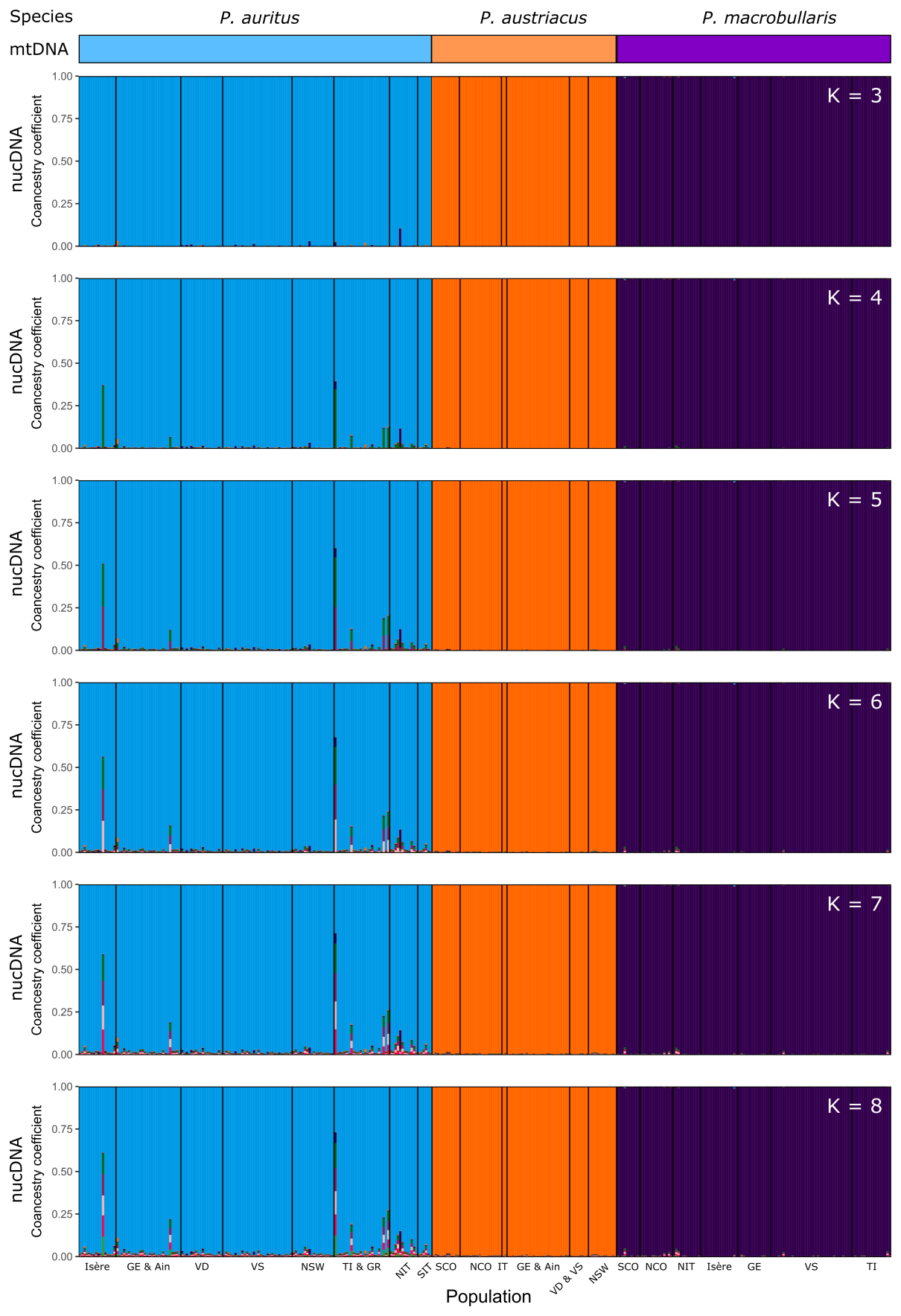


Appendix 7. Coancestry coefficients calculated by Structure for K = 2–5 from the microsatellite genotypes of 152 *P. auritus* individuals (15 loci). Ten independent replicates were performed, using correlated allele frequencies between genetic clusters. Each individual is represented by a vertical bar, and its coancestry coefficients estimated from nuclear genotypes are represented by colour portions of different sizes. Individuals are sorted by geographical region (separated by vertical black lines).


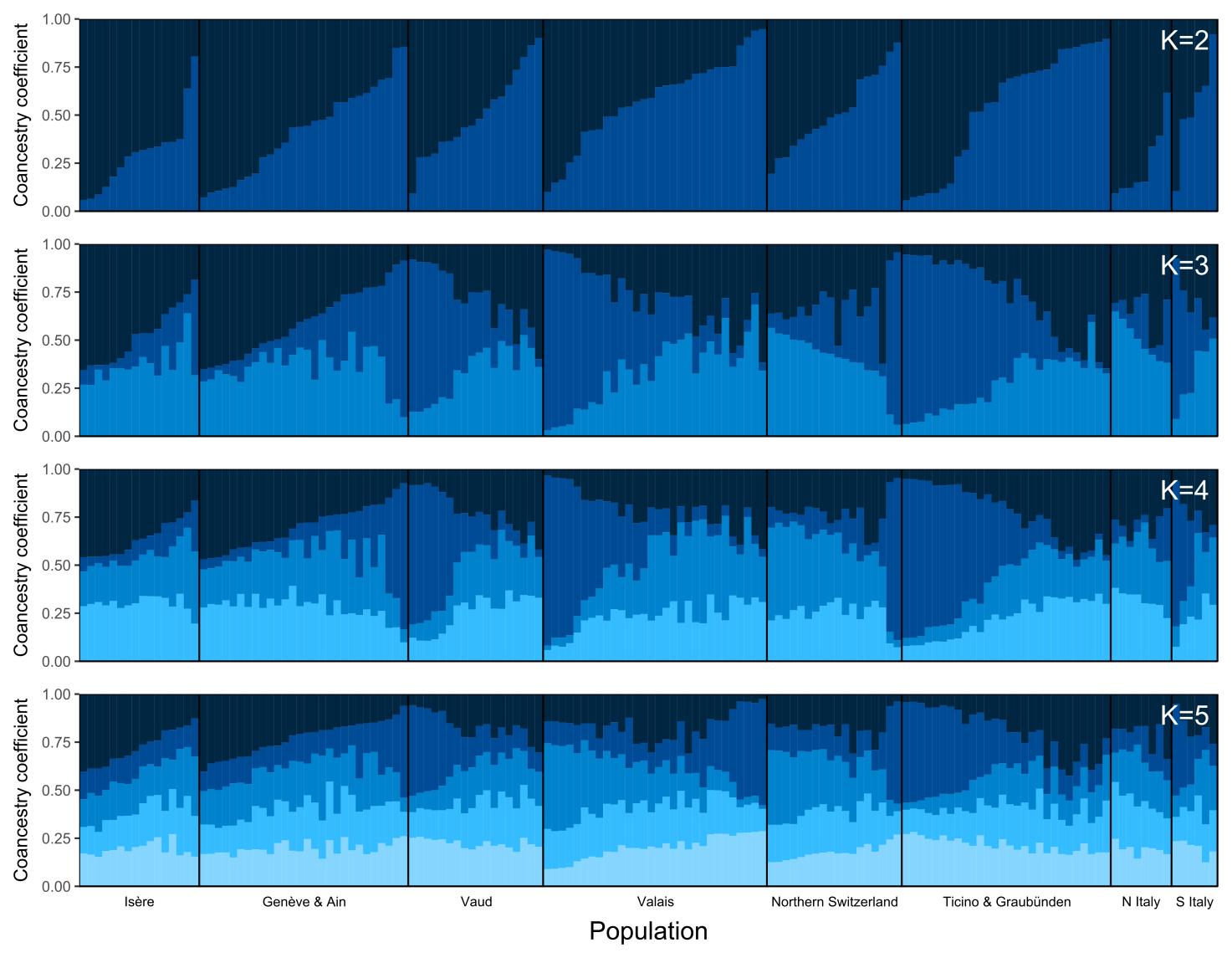


Appendix 8. Heat map of pairwise F_ST_ values (lower triangle) and associated p-value (upper triangle) calculated between populations of the three *Plecotus* species, on the basis of 15 microsatellite loci. * *P* < 0.05; ** *P* < 0.01; *** *P* < 0.001. Abbreviations for population names are found in the legend of Appendix 6.

|  |  | Isère | GE & Ain | VD | VS | NSW | TI & GR | NIT | SIT | SCO | NCO | IT | GE & Ain | VD & VS | NSW | SCO | NCO | NIT | Isère | GE | VS | TI |
| --- | --- | --- | --- | --- | --- | --- | --- | --- | --- | --- | --- | --- | --- | --- | --- | --- | --- | --- | --- | --- | --- | --- |
| *P. auritus* | Isère |  | NS | * | *** | NS | ** | NS | NS | *** | *** | ** | *** | *** | *** | *** | *** | *** | *** | *** | *** | *** |
|  | GE & Ain | 0.006 |  | * | *** | NS | *** | NS | NS | *** | *** | ** | *** | *** | *** | *** | *** | *** | *** | *** | *** | *** |
|  | VD | 0.009 | 0.008 |  | *** | NS | *** | NS | NS | *** | *** | *** | *** | *** | *** | *** | *** | *** | *** | *** | *** | *** |
|  | VS | 0.023 | 0.012 | 0.018 |  | *** | *** | NS | NS | *** | *** | * | *** | *** | *** | *** | *** | *** | *** | *** | *** | *** |
|  | NSW | 0.008 | 0.002 | 0.009 | 0.014 |  | *** | NS | NS | *** | *** | ** | *** | *** | *** | *** | *** | *** | *** | *** | *** | *** |
|  | TI & GR | 0.014 | 0.020 | 0.018 | 0.024 | 0.021 |  | NS | NS | *** | *** | *** | *** | *** | *** | *** | *** | *** | *** | *** | *** | *** |
|  | NIT | 0.000 | 0.000 | 0.000 | 0.000 | 0.000 | 0.002 |  | NS | *** | *** | * | *** | *** | *** | *** | *** | *** | *** | *** | *** | *** |
|  | SIT | 0.000 | 0.000 | 0.000 | 0.000 | 0.002 | 0.000 | 0.029 |  | *** | *** | * | *** | *** | *** | *** | *** | *** | *** | *** | *** | *** |
| *P. austriacus* | SCO | 0.156 | 0.173 | 0.188 | 0.202 | 0.194 | 0.164 | 0.148 | 0.140 |  | NS | NS | *** | *** | *** | *** | *** | *** | *** | *** | *** | *** |
|  | NCO | 0.177 | 0.191 | 0.205 | 0.220 | 0.209 | 0.186 | 0.175 | 0.168 | 0.000 |  | NS | *** | *** | *** | *** | *** | *** | *** | *** | *** | *** |
|  | IT | 0.169 | 0.193 | 0.212 | 0.236 | 0.225 | 0.190 | 0.220 | 0.251 | 0.033 | 0.031 |  | * | * | * | * | * | *** | *** | ** | *** | * |
|  | GE & Ain | 0.182 | 0.198 | 0.214 | 0.226 | 0.217 | 0.192 | 0.189 | 0.183 | 0.037 | 0.041 | 0.067 |  | NS | *** | *** | *** | *** | *** | *** | *** | *** |
|  | VD & VS | 0.168 | 0.188 | 0.205 | 0.214 | 0.207 | 0.179 | 0.170 | 0.164 | 0.043 | 0.052 | 0.056 | 0.010 |  | * | *** | *** | *** | *** | *** | *** | *** |
|  | NSW | 0.200 | 0.215 | 0.230 | 0.251 | 0.245 | 0.204 | 0.207 | 0.201 | 0.057 | 0.076 | 0.143 | 0.029 | 0.020 |  | *** | *** | *** | *** | *** | *** | *** |
| *P. macrobullaris* | SCO | 0.192 | 0.200 | 0.199 | 0.218 | 0.224 | 0.197 | 0.193 | 0.230 | 0.309 | 0.324 | 0.408 | 0.329 | 0.332 | 0.375 |  | NS | *** | *** | *** | *** | *** |
|  | NCO | 0.197 | 0.195 | 0.205 | 0.210 | 0.221 | 0.198 | 0.174 | 0.213 | 0.305 | 0.322 | 0.399 | 0.329 | 0.332 | 0.374 | 0.000 |  | *** | *** | *** | *** | *** |
|  | NIT | 0.153 | 0.161 | 0.165 | 0.166 | 0.179 | 0.164 | 0.157 | 0.211 | 0.282 | 0.302 | 0.381 | 0.302 | 0.304 | 0.349 | 0.078 | 0.103 |  | NS | NS | NS | NS |
|  | Isère | 0.199 | 0.197 | 0.204 | 0.202 | 0.216 | 0.202 | 0.177 | 0.225 | 0.315 | 0.334 | 0.412 | 0.334 | 0.343 | 0.385 | 0.097 | 0.108 | 0.019 |  | *** | * | NS |
|  | GE | 0.190 | 0.197 | 0.203 | 0.208 | 0.217 | 0.202 | 0.196 | 0.236 | 0.310 | 0.329 | 0.412 | 0.330 | 0.340 | 0.380 | 0.152 | 0.164 | 0.018 | 0.070 |  | *** | *** |
|  | VS | 0.218 | 0.219 | 0.230 | 0.228 | 0.247 | 0.228 | 0.203 | 0.240 | 0.324 | 0.339 | 0.403 | 0.338 | 0.350 | 0.387 | 0.097 | 0.117 | 0.014 | 0.023 | 0.065 |  | ** |
|  | TI | 0.183 | 0.186 | 0.192 | 0.198 | 0.207 | 0.191 | 0.164 | 0.207 | 0.299 | 0.318 | 0.395 | 0.319 | 0.326 | 0.366 | 0.091 | 0.106 | 0.001 | 0.014 | 0.028 | 0.022 |  |

**Appendix 9.** Coancestry coefficients calculated by Structure for K = 2–5 from the microsatellite genotypes of 79 *P. austriacus* individuals (19 loci). Ten independent replicates were performed, using correlated allele frequencies between genetic clusters. Each individual is represented by a vertical bar, and its coancestry coefficients estimated from nuclear genotypes are represented by colour portions of different sizes. Individuals are sorted by geographical region (separated by vertical black lines).


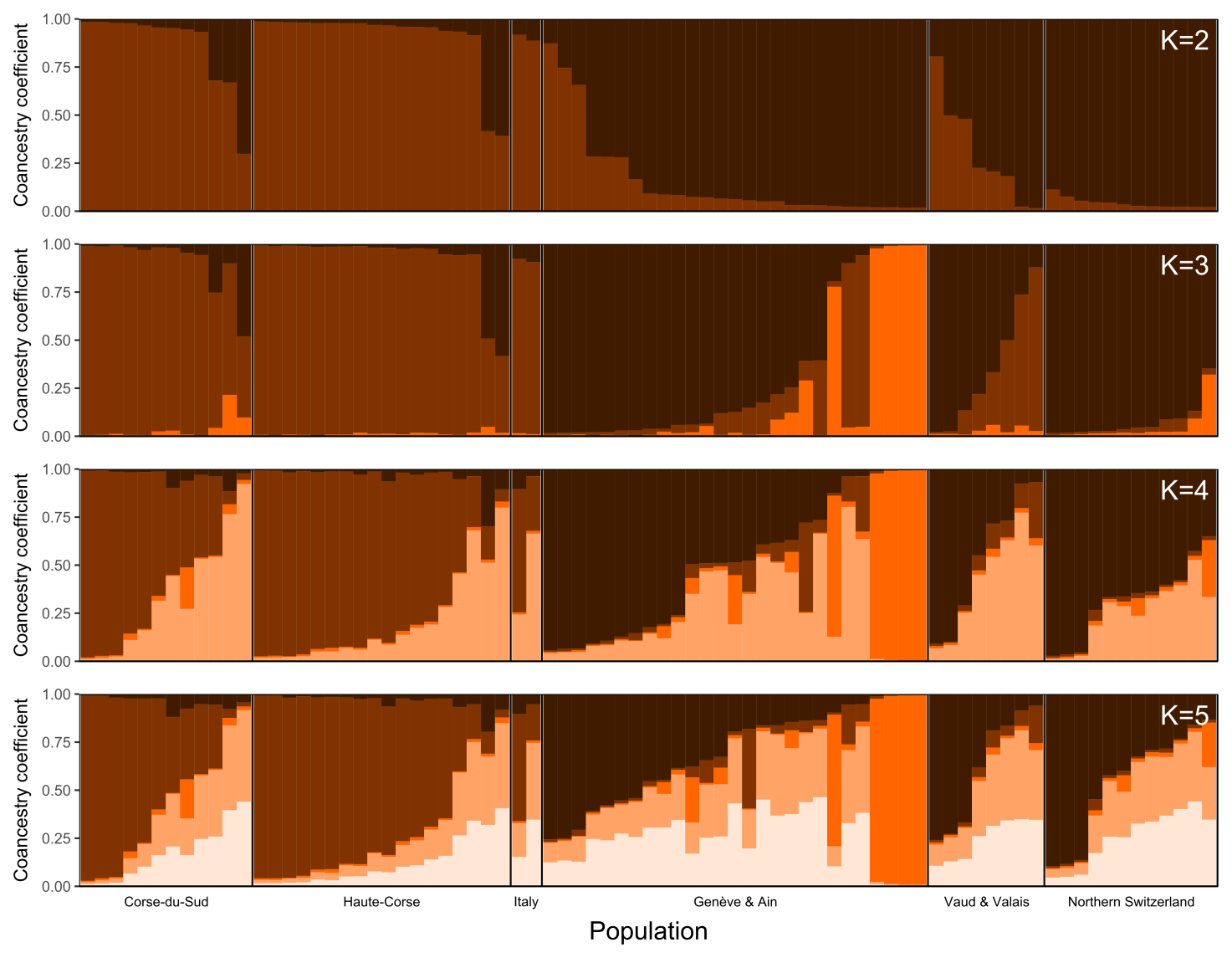


Appendix 10. Coancestry coefficients calculated by Structure for K = 2–5 from the microsatellite genotypes of 117 *P. macrobullaris* individuals (17 loci). Ten independent replicates were performed, using correlated allele frequencies between genetic clusters. Each individual is represented by a vertical bar, and its coancestry coefficients estimated from nuclear genotypes are represented by colour portions of different sizes. Individuals are sorted by geographical region (separated by vertical black lines).


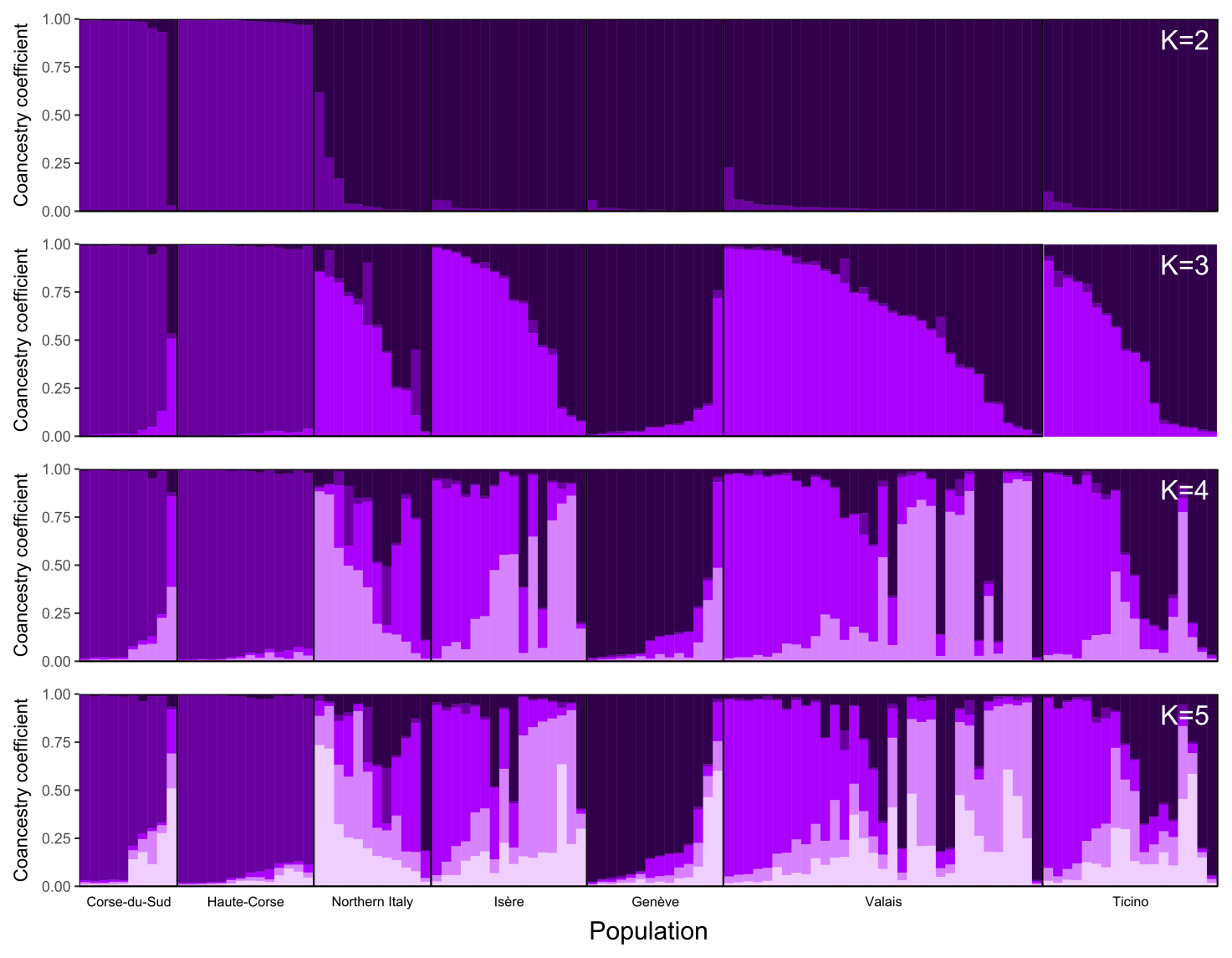


Appendix 11. Distribution map of the main mitochondrial lineages identified in *P. auritus* across its distribution. The dashed line delineates the distribution of the species based on Hutson *et al.* (2008). Geolocated individuals are represented by different combination of shades and shapes according to their mitochondrial lineage (Spitzenberger *et al.*, 2001, Kiefer *et al.*, 2002, Pestano *et al.*, 2003, Juste *et al.*, 2004, Veith *et al.*, 2004, Ibáñez *et al.*, 2006, Spitzenberger *et al.*, 2006, Kiefer, 2007, Galimberti *et al.*, 2012, Kruskop *et al.*, 2012, Çoraman *et al.*, 2013, Bogdanowicz *et al.*, 2015, Andriollo & Ruedi, 2018). The Sardinian lineage does not represent *P. sardus*, but the local lineage of *P. auritus*. Note that this Sardinian lineage was identified as distinct from Eastern and Western ones on the basis of the 16S marker, while the Abruzzo lineage was distinguished from the other two on the basis of the COI gene: therefore, the geographically close Sardinian and Abruzzo lineages could represent the two sides of the same coin. However, we consider here these two lineages are distinct from each other, since the Sardinian lineage seems genetically closer to the Western one and the Abruzzo lineage closer to the Eastern one on the basis of the sequences available.


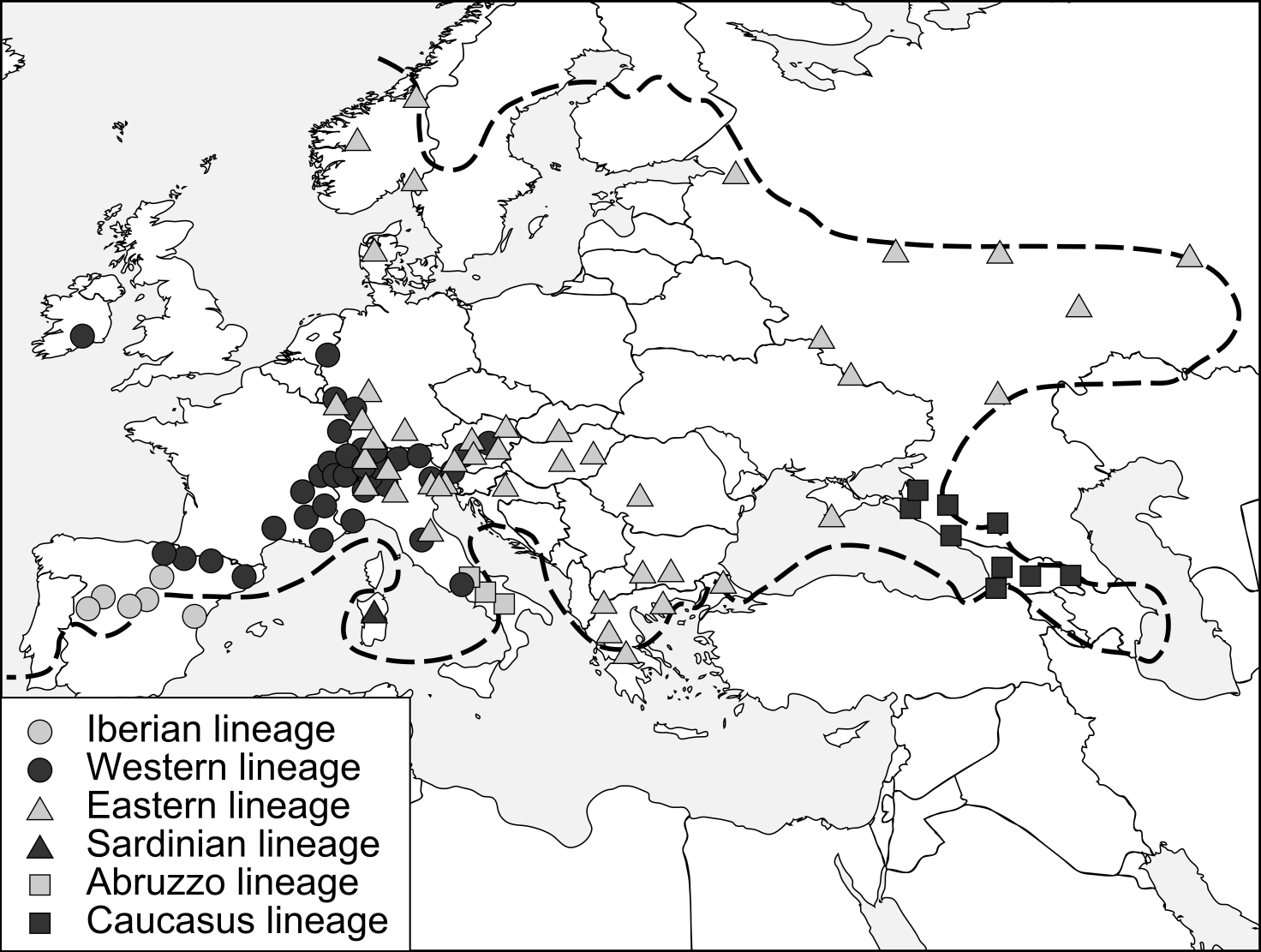


**References for the appendices**

Andriollo, T. & Ruedi, M. 2018. Novel molecular tools to identify *Plecotus* bats in sympatry and a review of their distribution in Switzerland. *Revue suisse de Zoologie* 125: 61-72. https://doi.org/10.5281/zenodo.1196013.

Bogdanowicz, W., Hulva, P., Černá Bolfíková, B., Buś, M. M., Rychlicka, E., Sztencel-Jabłonka, A., Cistrone, L. & Russo, D. 2015. Cryptic diversity of Italian bats and the role of the Apennine refugium in the phylogeography of the Western Palaearctic. *Zoological Journal of the Linnean Society* 174: 635-648. https://doi.org/10.1111/zoj.12248.

Çoraman, E., Furman, A., Karataş, A. & Bilgin, R. 2013. Phylogeographic analysis of Anatolian bats highlights the importance of the region for preserving the Chiropteran mitochondrial genetic diversity in the Western Palaearctic. *Conservation Genetics* 14: 1205-1216. https://doi.org/10.1007/s10592-013-0509-4.

Galimberti, A., Spada, M., Russo, D., Mucedda, M., Agnelli, P., Crottini, A., Ferri, E., Martinoli, A. & Casiraghi, M. 2012. Integrated operational taxonomic units (IOTUs) in echolocating bats: a bridge between molecular and traditional taxonomy. *PLoS ONE* 7. https://doi.org/10.1371/journal.pone.0040122.

Hutson, A. M., Spitzenberger, F., Aulagnier, S., Coroiu, I., Karataş, A., Juste, J., Paunović, M., Palmeirim, J. & Benda, P. 2008. *Plecotus auritus*. *The IUCN Red List of Threatened Species 2008*. 10.2305/IUCN.UK.2008.RLTS.T17596A7154745.en.

Ibáñez, C., García-Mudarra, J. L., Ruedi, M., Stadelmann, B. & Juste, J. 2006. The Iberian contribution to cryptic diversity in European bats. *Acta Chiropterologica* 8: 277-297. https://doi.org/10.3161/1733-5329(2006)8[277:TICTCD]2.0.CO;2.

Juste, J., Ibàñez, C., Muñoz, J., Trujillo, D., Benda, P., Karatas, A. & Ruedi, M. 2004. Mitochondrial phylogeography of the long-eared bats (*Plecotus*) in the Mediterranean Palaearctic and Atlantic islands. *Molecular Phylogenetics and Evolution* 31: 1114-1126. https://doi.org/10.1016/j.ympev.2003.10.005.

Kiefer, A. 2007. Phylogeny of Western Palaearctic long-eared bats (Mammalia, Chiroptera, *Plecotus*) – a molecular perspective. Johannes Gutenberg-Universität in Mainz, pp. 132. https://d-nb.info/98983185X/34 (accessed on 23.07.2018).

Kiefer, A., Mayer, F., Kosuch, J., von Helversen, O. & Veith, M. 2002. Conflicting molecular phylogenies of European long-eared bats (*Plecotus*) can be explained by cryptic diversity. *Molecular Phylogenetics and Evolution* 25: 557-566. https://doi.org/10.1016/S1055-7903(02)00299-3.

Kruskop, S. V., Borisenko, A. V., Ivanova, N. V., Lim, B. K. & Eger, J. L. 2012. Genetic diversity of northeastern Palaearctic bats as revealed by DNA barcodes. *Acta Chiropterologica* 14: 1-14. https://doi.org/10.3161/150811012x654222.

Pestano, J., Brown, R., Suarez, N., Benzal, J. & Fajardo, S. 2003. Intraspecific evolution of Canary Island Plecotine bats, based on mtDNA sequences. *Heredity* 90: 302-307. https://doi.org/10.1038/sj.hdy.6800240.

Spitzenberger, F., Pialek, J. & Haring, E. 2001. Systematics of the genus *Plecotus* (Mammalia, Vespertilionidae) in Austria based on morphometric and molecular investigations. *Folia Zoologica* 50: 161-172.

Spitzenberger, F., Strelkov, P., Winkler, H. & Haring, E. 2006. A preliminary revision of the genus *Plecotus* (Chiroptera, Vespertilionidae) based on genetic and morphological results. *Zoologica Scripta* 35: 187-230. https://doi.org/10.1111/j.1463-6409.2006.00224.x.

Veith, M., Beer, N., Kiefer, A., Johannesen, J. & Seitz, A. 2004. The role of swarming sites for maintaining gene flow in the brown long-eared bat (*Plecotus auritus*). *Heredity* 93: 342-349. https://doi.org/10.1038/sj.hdy.6800509.
